# Partial loss-of-function mutations in *GINS4* lead to NK cell deficiency with neutropenia

**DOI:** 10.1172/jci.insight.154948

**Published:** 2022-11-08

**Authors:** Matilde I. Conte, M. Cecilia Poli, Angelo Taglialatela, Giuseppe Leuzzi, Ivan K. Chinn, Sandra A. Salinas, Emma Rey-Jurado, Nixa Olivares, Liz Veramendi-Espinoza, Alberto Ciccia, James R. Lupski, Juan Carlos Aldave Becerra, Emily M. Mace, Jordan S. Orange

**Affiliations:** 1 Department of Pediatrics, Vagelos College of Physicians and Surgeons, Columbia University Irving Medical Center, New York, New York, USA.; 2 Faculty of Medicine, Clínica Alemana Universidad del Desarrollo, Santiago, Chile.; 3 Immunology and Rheumatology Unit, Hospital Roberto del Rio, Santiago, Chile.; 4 Department of Genetics and Development, Herbert Irving Comprehensive Cancer Center, Columbia University Irving Medical Center, New York, New York, USA.; 5 Department of Pediatrics, Baylor College of Medicine, Houston, Texas, USA.; 6 Division of Immunology, Allergy, and Retrovirology, Texas Children’s Hospital, Houston, Texas, USA.; 7 Allergy and Clinical Immunology, Hospital Nacional Edgardo Rebagliati Martins, Lima, Peru.; 8 Department of Molecular and Human Genetics, Baylor College of Medicine, Houston, Texas, USA.

**Keywords:** Cell Biology, Immunology, Cell cycle, Monogenic diseases, NK cells

## Abstract

Human NK cell deficiency (NKD) is a primary immunodeficiency in which the main clinically relevant immunological defect involves missing or dysfunctional NK cells. Here, we describe a familial NKD case in which 2 siblings had a substantive NKD and neutropenia in the absence of other immune system abnormalities. Exome sequencing identified compound heterozygous variants in Go-Ichi-Ni-San (GINS) complex subunit 4 (*GINS4*, also known as *SLD5*), an essential component of the human replicative helicase, which we demonstrate to have a damaging impact upon the expression and assembly of the GINS complex. Cells derived from affected individuals and a *GINS4*-knockdown cell line demonstrate delayed cell cycle progression, without signs of improper DNA synthesis or increased replication stress. By modeling partial *GINS4* depletion in differentiating NK cells in vitro, we demonstrate the causal relationship between the genotype and the NK cell phenotype, as well as a cell-intrinsic defect in NK cell development. Thus, biallelic partial loss-of-function mutations in *GINS4* define a potentially novel disease-causing gene underlying NKD with neutropenia. Together with the previously described mutations in other helicase genes causing NKD, and with the mild defects observed in other human cells, these variants underscore the importance of this pathway in NK cell biology.

## Graphical abstract

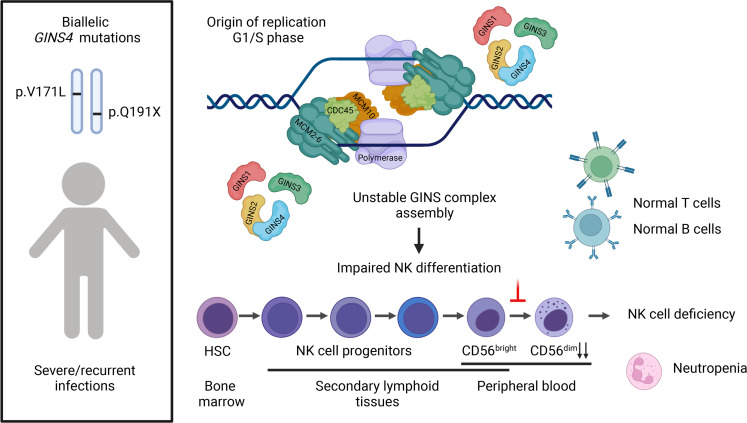

## Introduction

NK cells are lymphocytes of the innate immune system that critically function in targeting virally infected and tumorigenic cells. They exert rapid cytotoxic functions by forming synaptic contacts with susceptible target cells and secreting effector molecules, including granzymes and perforin contained in preformed lytic granules, at the lytic immune synapse (1). As components of the innate immune system, NK cells express germline-encoded activating and inhibitory receptors whose balance modulates their capability to either spare healthy or kill diseased cells (2).

In humans, NK cells are typically identified as CD3^−^ and CD56^+^ lymphocytes comprising 5%–20% of those in peripheral blood, and they populate primary and secondary lymphoid organs, as well as peripheral tissues (3). NK cells are highly heterogeneous, although 2 major subsets in peripheral blood are classically described as CD3^−^CD56^bright^CD16^−^ and CD3^−^CD56^dim^CD16^+^, each having different functional properties. CD56^bright^ cells potently produce cytokines and are more frequently found in tissues, whereas CD56^dim^ cells excel in cytotoxic functions and represent approximately 90% of circulating NK cells (4). NK cells originate from hematopoietic stem cells (HSC) in BM and undergo terminal maturation in secondary lymphoid tissues. CD56^bright^ cells can be direct precursors of CD56^dim^ NK cells as demonstrated by their: (a) having longer telomeres (5); (b) appearing more rapidly after BM transplantation (6); (c) being able to transition in vitro to CD56^dim^ (7); and (d) developing into CD16^+^ NK cells in humanized immune mice (8). Only BM is essential for the generation of NK cells, but their development is shaped by the secondary lymphoid tissue microenvironment (9).

NK cell deficiency (NKD) is a primary immunodeficiency in which NK cells are nonfunctional, missing, or have dysregulated terminal maturation (10) and in which NK cell abnormalities account for the majority of the clinically relevant immunological defects. NKD has the striking clinical hallmark of unusual susceptibility to severe and atypical manifestations of herpesviruses, including cytomegalovirus (CMV), varicella zoster virus (VZV), herpes simplex virus (HSV), EBV, and/or problems with papilloma viruses, highlighting the importance of NK cells for host defense (10–12). The study of patients with NKD provides a meaningful opportunity to understand the complex process and requirements of human NK cell development and function.

Currently, 7 NKD-causing monogenic conditions have been described due to pathogenic variants in the following genes: GATA binding domain protein 2 (*GATA2*), IFN regulatory factor 8 (*IRF8*), Fc fragment of IgG receptor IIIa (*FCGR3A*), regulator of telomere elongation helicase I (*RTEL1*), minichromosome maintenance complex component 4 (*MCM4*), Go-Ichi-Ni-San (GINS) complex subunit 1 (*GINS1*), and minichromosome maintenance 10 replication initiation factor (*MCM10*) (13–21). NKD caused by *FCGR3A* variants is referred to as a “functional NKD,” as only NK cell function is impaired, with normal NK cell maturation and development. The other 6 described NKD conditions can be labeled “classical NKD” and are defined by low or absent NK cell numbers or abnormal distribution of NK cell subsets, consistent with impaired NK cell development or maturation.

Of note, 3 of the 6 classical NKDs are caused by impactful variants in genes that comprise the replicative DNA helicase, underscoring the importance of this complex and, more broadly, the regulation of DNA replication and the cell cycle in human NK cell development. *MCM4* deficiency leads to reduced NK cells in the periphery, with a specific reduction of the CD56^dim^ NK cell subset and concomitant overrepresentation of the CD56^bright^ subset (15). While NKD is the major immunological manifestation of human MCM4 deficiency, the clinical phenotype of affected individuals also includes short stature, adrenal insufficiency, and microcephaly (15, 17, 22). Similarly, rare and damaging biallelic variants in *GINS1* cause short stature, dysmorphic features, and decreased NK cell frequencies with an increased CD56^bright^/CD56^dim^ ratio. G*INS1* cases represent the originally described familial NKD and, additionally, have chronic neutropenia that can be corrected with the administration of G-CSF (14, 23, 24). As predicted by their cellular function, partial loss-of-function (LoF) mutations in *MCM4* and *GINS1* result in impaired DNA replication with increased DNA damage. In the case of MCM4 deficiency, increased apoptosis in the CD56^bright^ NK cell subset was reversible by treatment with IL-2 (15). However, these CD56^bright^ NK cells did not proliferate at the same rate as unaffected control cells in response to stimulation with IL-2. This finding suggested that the accumulation of chromosomal aberrations during proliferation of the CD56^bright^ subset leads to the generation of only a few CD56^dim^ NK cells, strengthening the preexisting hypothesis that CD56^dim^ NK cells arise from the CD56^bright^ subset.

Recently, NKD was reported due to biallelic variants in *MCM10* and characterized by a near absence of NK cells in the proband with increased frequency of CD56^bright^ NK cells (19). These variants cause replication stress (RS), increased genomic instability, and telomere maintenance defects in multiple cell types; however, the predominant immune defect consisted of NKD (19, 25). Recapitulation of NK cell development using MCM10-knockdown (MCM10-KD) CD34^+^ cells, and patient-derived iPSCs in a humanized mouse model, demonstrated that loss of MCM10 function leads to impaired NK cell maturation and decreased survival of NK cells, further pointing to the reliance of NK cell development upon the replicative DNA helicase (19).

The human CMG helicase (CDC45, MCM2-7, and GINS1-4) complex is essential for dsDNA unwinding at the replication fork. The MCM2-7 ring is the core helicase component and is loaded as an inactive double hexamer at thousands of replication origins during the G1 phase in a process called “origin licensing” (26). The activation of MCM2-7 occurs during the early S phase and is tightly regulated by recruiting the firing factors CDC45 and the GINS1-4 tetramer. For proper initiation and elongation of DNA synthesis, the association of MCM10, CTF4, and subsequent DNA polymerases to the CMG is required (27). Inactive MCM2-7 complexes are usually expressed in excess compared with the number of replication forks formed in S phase and are distributed at locations distant from the origins, presenting a paradox called the “MCM paradox” (28). In addition to supplying backup origins under RS, inactive origins serve to function in fork speed management in order to prevent genome instability and tumor formation (29). The mechanism by which biallelic partial LoF mutations in replicative helicase genes uniquely leads to NK cell abnormalities — and neutropenia, in the case of GINS1 deficiency — while sparing most other immune cells is poorly understood. Here, we describe a case of biallelic loss of another CMG helicase component, *GINS4*, resulting in NKD with neutropenia. By cellular modeling of the *GINS4* variants, we validate the damaging impact of both variants on GINS complex expression and stability and establish a requirement for GINS4 in NK cell development.

## Results

### Clinical presentation and immunological phenotype.

Two siblings, born to nonconsanguineous healthy parents, presented with suspected immunodeficiency. The proband (II.1) had intrauterine growth restriction, growth delay, tonsillar hypertrophy, cryptorchidism, and localized BCG infection after vaccination. At 11 months of age, he developed generalized seizures with intracranial calcifications, suggesting a history of CMV infection; however, outside of elevated CMV-IgG, additional clinical studies were not performed at that time. Subsequently, within the first 3 years of life, he developed pneumonia, sinusitis, intermittent diarrhea, gastrointestinal sepsis, oral abscess, gingivitis, recurrent otitis, and severe varicella infection with a necrotizing nasolabial ulcer. Currently, he continues to have intermittent diarrhea and recurrent herpes labialis. The proband’s younger sister (II.2) had experienced non–life-threatening varicella at 10 months of age and has recurrent herpes labialis (Table 1).

Individuals II.1 and II.2 had normal T and B cell counts, low NK cell counts, normal or increased immunoglobulin levels, and neutropenia (more substantive in individual II.1) (Supplemental Table 1; supplemental material available online with this article; https://doi.org/10.1172/jci.insight.154948DS1). BM analysis in the proband showed incomplete neutrophil maturation. He was successfully treated with G-CSF starting at 3 years of age, initially daily and currently twice a week, resulting in some clinical improvement of gingivitis, fever, and diarrhea. His sister was treated monthly with G-CSF starting at 7 months of age and for 2 years. She is no longer receiving any treatment and has stable moderate neutrophil counts. The proband’s parents were largely unaffected, although the mother has recurrent herpes labialis. The proband’s maternal uncle died from severe gastrointestinal sepsis at 9 months of age.

**Figure 1:**
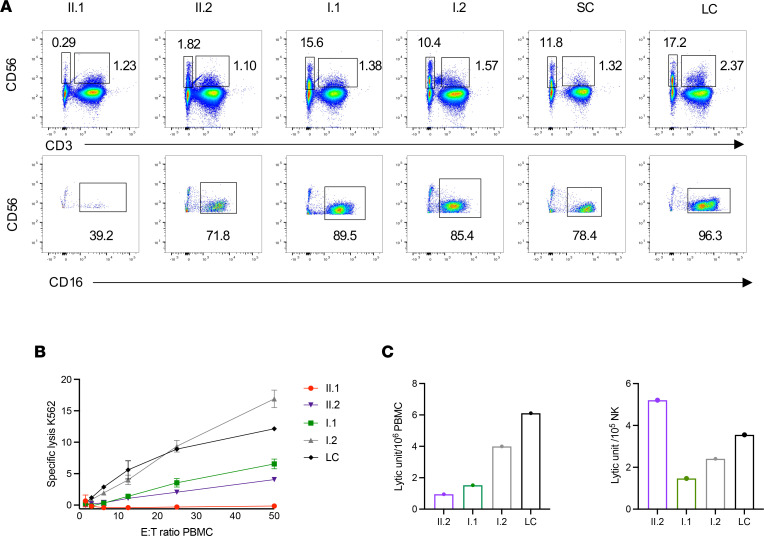
Frequency and cytotoxic function of peripheral NK cells. (**A**) Flow cytometric analysis of CD56^+^CD3^−^ NK cells and CD56^+^CD3^+^ NKT (top) and frequency of CD16^+^ NK cells (bottom). Two healthy donors, siblings (II.1, II.2), and their parents (I.1, I.2) are shown. Data are from a representative experiment of 3 independent repeats summarized in Table 2. LC, laboratory control; SC, shipping control. (**B**) ^51^Cr release assay to evaluate the cytotoxic function against K562 is shown for siblings, healthy parents, and LC. Data are shown as mean ± SD of technical replicates. Data are from a representative experiment of 2 independent repeats. (**C**) Graph of the lytic units calculated from the values of the ^51^Cr release assay shown in **B**.

Given the viral infections and low NK cell counts, research-level evaluations were performed. Flow cytometry analysis demonstrated consistent NK cell abnormalities in both individuals II.1 and II.2. The frequency of CD56^+^CD3^−^ NK cells was substantially decreased, with values ranging from 0.002% to 1.34 % in individual II.1 and from 0.1% to 3.01% in individual II.2, with an increased proportion of the CD56^bright^ NK subset and decreased CD16^+^ NK cells (Figure 1A and Table 2). We further evaluated the expression of the NK cell maturation markers CD57 and CD94. Lower MFI for both was observed at baseline in both siblings’ NK cells, as well in their CD56^dim^ and CD56^bright^ subsets. Interestingly, the expression of CD94 and CD57 did not increase on NK cells after stimulation with IL-15 for 48 hours (Supplemental Figure 1A).

We also evaluated NK cells for the expression of the effector molecules perforin and IFN-γ and CD107a as an indicator of activation and degranulation upon stimulation of PBMC with phorbol 12-myristate 13-acetate ionomycin (PMA-iono). The levels of CD107a and perforin expression per NK cell were seemingly normal in both individuals. The IFN-γ production, however, was lower in II.2 than in the HD, despite an increased frequency of NK cells (3%) compared with previous evaluations (Supplemental Figure 1B). Notably, survival of the proband’s NK cells upon stimulation with PMA was reduced.

To evaluate NK cell cytotoxic function, chromium 51 (^51^Cr) release assays were performed for all family members and were compared with that of healthy donors (HDs). Very low lytic activity against K562 target cells was identified in II.1 and II.2 (Figure 1B), which was not rescued by adding IL-2 to the assay (data not shown). Their mother (I.1) also had reduced NK cell cytotoxic function, despite seemingly normal frequencies of NK cells. The cytotoxic activity was still reduced when expressed as lytic unit (LU) per PBMCs (Figure 1C, left panel). This difference was no longer present in individual II.2 when we normalized for the extreme underrepresentation of NK cells to define the LU per NK cells; thus, the impaired cytotoxic activity is likely attributed to the lower frequency of NK cells in this individual. The normalized analysis, however, identifies lower LU per NK cells in individual I.1, the proband’s mother (Figure 1C, right panel).

The limited availability of primary samples prevented extensive immunological evaluation; nevertheless, we investigated some circulating T cell subsets. Individuals II.1 and II.2 had normal numbers and percentages of naive and memory CD4^+^ and CD8^+^ T cells (Supplemental Figure 2A). Also, mucosal-associated invariant T cells (MAIT) and γδ-T subsets were seemingly normal (Supplemental Figure 2B).

In aggregate, the clinical history of the proband in light of the NK cell phenotypic and functional data suggested the familial presence of immunodeficiency with a major defect in NK cell numbers and resulting lytic function.

### Compound heterozygous variants in GINS4 identified via exome sequencing.

**Figure 2:**
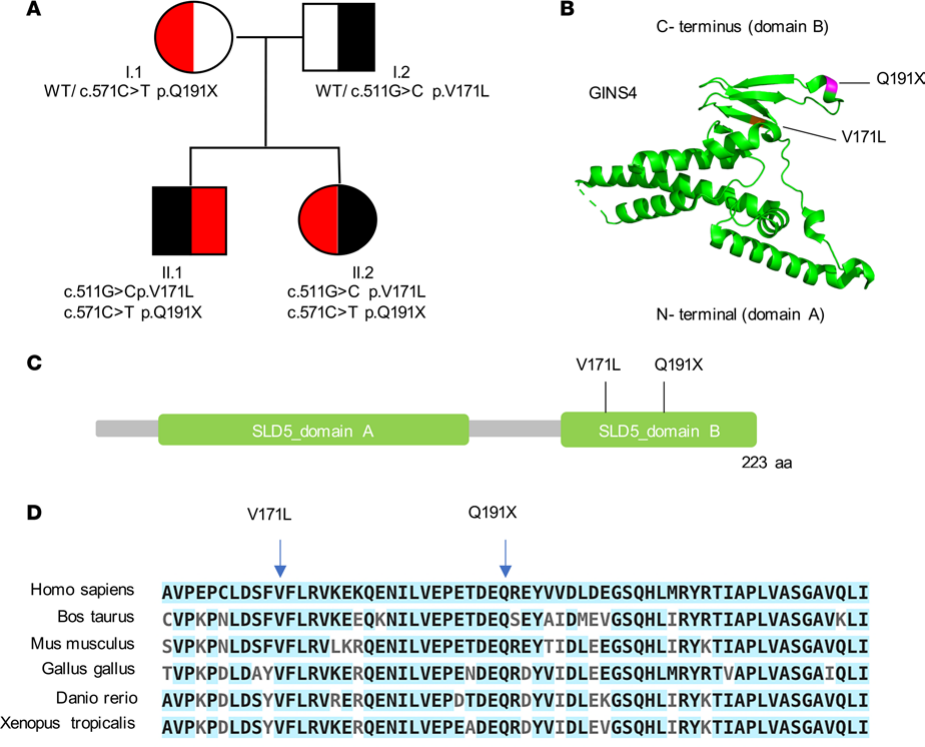
Identification of compound heterozygous variants in the *GINS4* gene by whole-exome sequencing. (**A**) Pedigree of the family denoting variants of *GINS4*. (**B**) GINS4 protein 3D structure prediction showing α-helices at N-terminus and B-strands at C-terminus (variants labeled in violet). (**C**) Schematic representation of GINS4 (SLD5) protein and variants mapping at the C-terminal domain. (**D**) Multiple protein sequence analysis using ClustalW shows evolutionary conservation of the C-terminal domain of GINS4. Conserved amino acids are highlighted in light blue. The position of both variants is indicated.

Exome sequencing on DNA from blood samples of the proband, his sister, and their parents was performed. Analyses were executed in accordance with dominant and recessive models of Mendelian inheritance. Given that both parents were clinically unaffected, we hypothesized a recessive model of inheritance. Poor quality and frequent variants (minor allelic frequency > 0.001) were filtered out, and focus was given to biallelic variants. The proband and his sister shared 2 heterozygous compound variants in exon 7 of *GINS4*, each inherited from an individual parent. These changes encompassed a missense variant encoding a valine-to-leucine change, NM_032336:exon7:c.511G>C:p.V171L, and a stop gain variant, NM_032336:exon7:c.C571T:p.Q191X (Figure 2A), both mapping to the C-terminal domain B of GINS4 (Figure 2, B and C). Segregation of both variants among the proband, sister, and parents was confirmed by Sanger sequencing (Supplemental Figure 3).

Bioinformatic analyses suggested that these variants are damaging and pathogenic. Specifically, both are in a highly conserved region (Figure 2D) and very rare; only 7 V171L and no Q191X alleles are reported in The Genome Aggregation Database (gnomAD v3.1.1). Using Combined Annotation Dependent Depletion (CADD) scores (30), the variants are predicted as damaging, with values of 26.4 (V171L) and 39 (Q191X) (for *GINS4*-specific mutation significance cutoff score, MSC of 5.720) (31). Moreover, a gene damage index (GDI) prediction of 1.022 suggests that *GINS4* is likely to be a disease-causing gene (32). A complete list of all prioritized variants identified is provided in Supplemental Table 2. Given the genetic model of inheritance, fulfillment of bioinformatic criteria, and literature demonstrating a connection between the DNA replicative helicase and NKD, we considered *GINS4* a potential candidate disease gene.

### Variants in GINS4 affect GINS complex expression and assembly.

**Figure 3:**
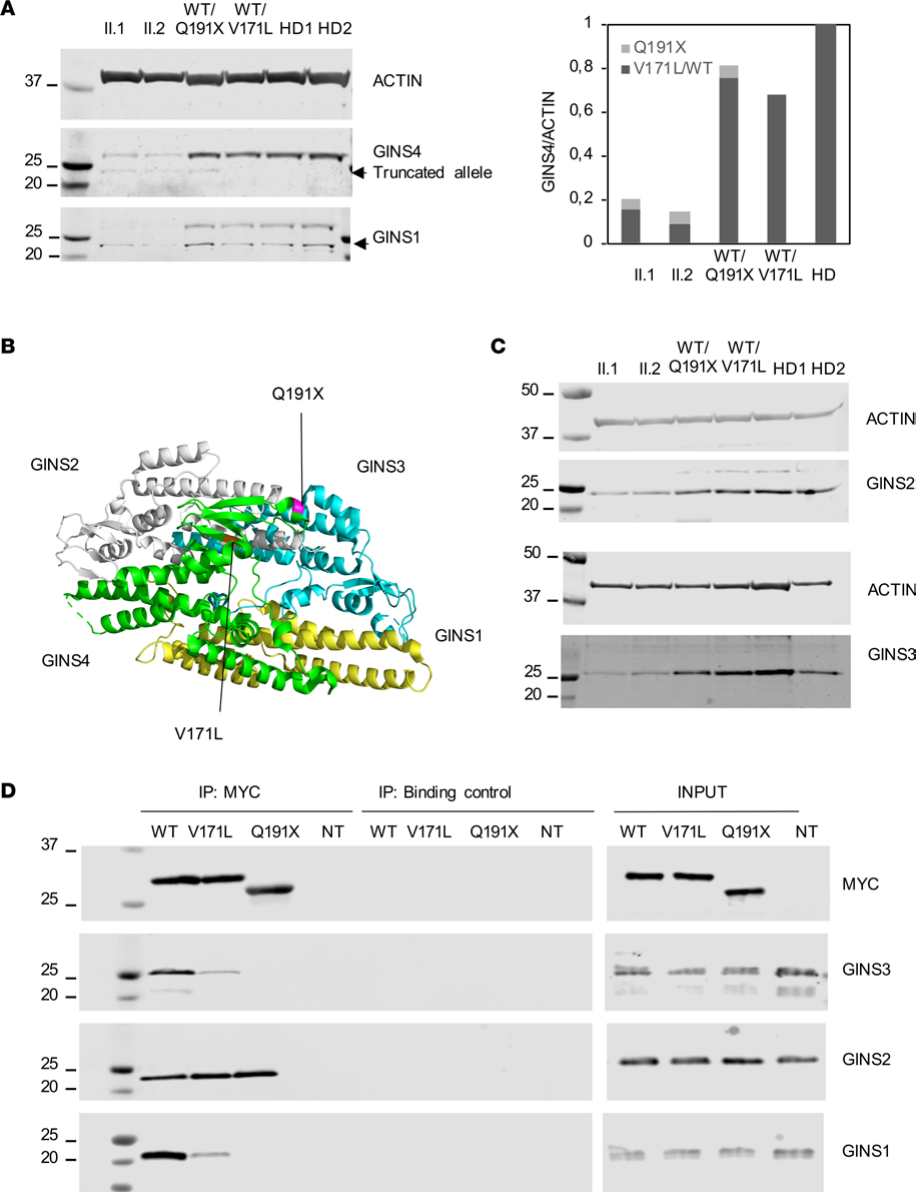
V171L and Q191X destabilize the expression of GINS4 and other GINS proteins and impair GINS complex assembly. (**A**) GINS4 and GINS1 protein expression in BLCL derived from siblings and parents compared with those from 2 healthy donors (HD). Relative GINS4 protein expression of family members and 2 HDs from 3 independent experiments. (**B**) GINS complex 3D structure showing the position of V171L and Q191X variants. (**C**) GINS2 and GINS3 protein expression in BLCL derived from siblings and parents compared with 2 HD. One representative experiment of 3 independent experiments is shown (**D**) HEK293T cells were transiently transfected with Myc-*GINS4* WT, Myc-*GINS4*-V171L, or Myc-GINS4 Q191X plasmids. After 48 hours, whole cellular extracts were immunoprecipitated with anti-MYC, and blots were probed for GINS1, GINS2, GINS3, and MYC. Data are representative of 3 independent experiments.

GINS4 is a small protein of 26 kDa consisting of 223 amino acids. To evaluate the potentially damaging impact of the variants, we first assessed protein expression. EBV-transformed B lymphoblastoid cell lines (BLCL) derived from peripheral blood of both the proband and his sister consistently expressed 15%–20% of the GINS4 protein found in the BLCL of HDs, underscoring the combined damaging effects of the variants that lead to the destabilization of the protein (Figure 3A). Both variants map at the C-terminal domain, and the premature stop codon Q191X maps 4 nucleotides upstream of the last exon-exon junction, which, according to the 50–55 nucleotide rule, should give rise to transcripts able to escape nonsense-mediated decay (NMD) (33). The truncated allele is predicted to result in a shorter protein of 23 kDa and was detectable by Western blot of BLCL lysates at the expected molecular size (Figure 3A).

Expression of Myc-*GINS4* WT and Myc-*GINS4* Q191X plasmids in HEK293T cells confirmed the specificity of the antibody used to detect GINS4 in BLCL and the ability to detect the shorter mutant isoform (Supplemental Figure 4A). To further evaluate the possibility that the stop codon allele can partially escape NMD, we sequenced the cDNA samples derived from the individual-derived BLCLs, which confirmed the presence of the stop codon transcript in both siblings and the mother, demonstrating at least the ongoing presence of the transcript for the truncation variant in physiologic material (Supplemental Figure 4B). In addition, the missense variant V171L appeared partially unstable at the protein level, as both siblings had consistent protein expression of the full-length variant ranging from 15% to 20%, lower than the expected 50% (Figure 3A). In line with this observation, the father carrying the single V171L heterozygous variant expressed 70%–75% of the protein (Figure 3A).

GINS4 is part of the GINS complex (Figure 3B), which is comprised of 4 subunits that are tightly associated and regulated (34). Crystal structures and electron microscopy of the GINS complex define a trapezoidal tetramer held by intersubunit hydrophobic bonds, in which GINS4 is directly bound to GINS2 through both its N-terminal and C-terminal domains and to GINS1 and GINS3 through its C-terminal domain. The N-terminal interaction between GINS2 and GINS4 is the most extensive interface in the complex (35). The crystal structure further indicates that the C-terminal residues of GINS4 are required for the assembly of the GINS core complex (36). Due to precedence for the level of GINS components impacting the expression of other GINS proteins (14, 37), we assessed the levels of GINS proteins by Western blot in BLCLs. Substantively lower levels of GINS1 and GINS3, and a slight reduction of GINS2 in cells from both II.1 and II.2, were observed (Figure 3, A and C).

To evaluate the contribution of each variant on the stability and formation of the GINS complex, independently of the proband genetic background, we transiently overexpressed the Myc-tagged versions of the V171L, Q191X, and WT alleles in HEK293T cells, coimmunoprecipitated them, and measured the levels of the other associated GINS proteins. While equivalent levels of Myc-tagged V171L, Q191X, and WT GINS4 were present in the total cell lysates, the missense and the stop codon containing GINS4 impaired GINS complex assembly (Figure 3D). Specifically, in Myc immunoprecipitates, GINS4 V171L retained reduced amounts of GINS1 and GINS3, and the amounts retained by GINS4 Q191X were nearly absent (Figure 3D). Interestingly, both V171L and Q191X GINS4 retained interaction with GINS2 (Figure 3D), likely mediated by their presumably unaffected N-terminal domain. Thus, both V171L and Q191X GINS4 variants impacted the stability of GINS4 and other GINS proteins and had a similar impact on impairing the core complex assembly, with the stop codon being more damaging.

### GINS4 variants are associated with mild delays in cell cycle progression.

The GINS complex is an essential component of the replicative helicase, and it is required for proper activation and progression of the DNA replication fork during the synthesis (S) phase of the cell cycle (38). In human cells, GINS levels correlate with cell proliferation, with the highest levels found in some of the most highly proliferating cells (39). A reduced amount of GINS4 and other GINS subunits (less than 10%) have been associated with increased frequency of cells in G1 and at the G1/S transition, suggesting an impaired entry and progression into S phase (39). This decrease is also associated with an accumulation of DNA double-strand breaks (DSB), presumably induced by improper DNA replication (39). However, it has been reported that hypomorphic effects of GINS4 depletion result in an accumulation of cells in the prometaphase stage of mitosis because of spindle pole disorganization (40). Those investigators did not observe increased DNA damage or delayed entry in S phase, suggesting that the arrest in G2/M is not a consequence of increased DNA damage and highlighting additional mitotic functions of GINS4.

Since these multiple roles of GINS4 in cell cycle phases have been described, we assessed cell cycle progression, replication fork stability, and DNA damage in BLCLs from the proband and his sister. For comparison, we analyzed the cell cycle in BLCLs derived from family members (2 healthy parents) and 2 HDs.

**Figure 4:**
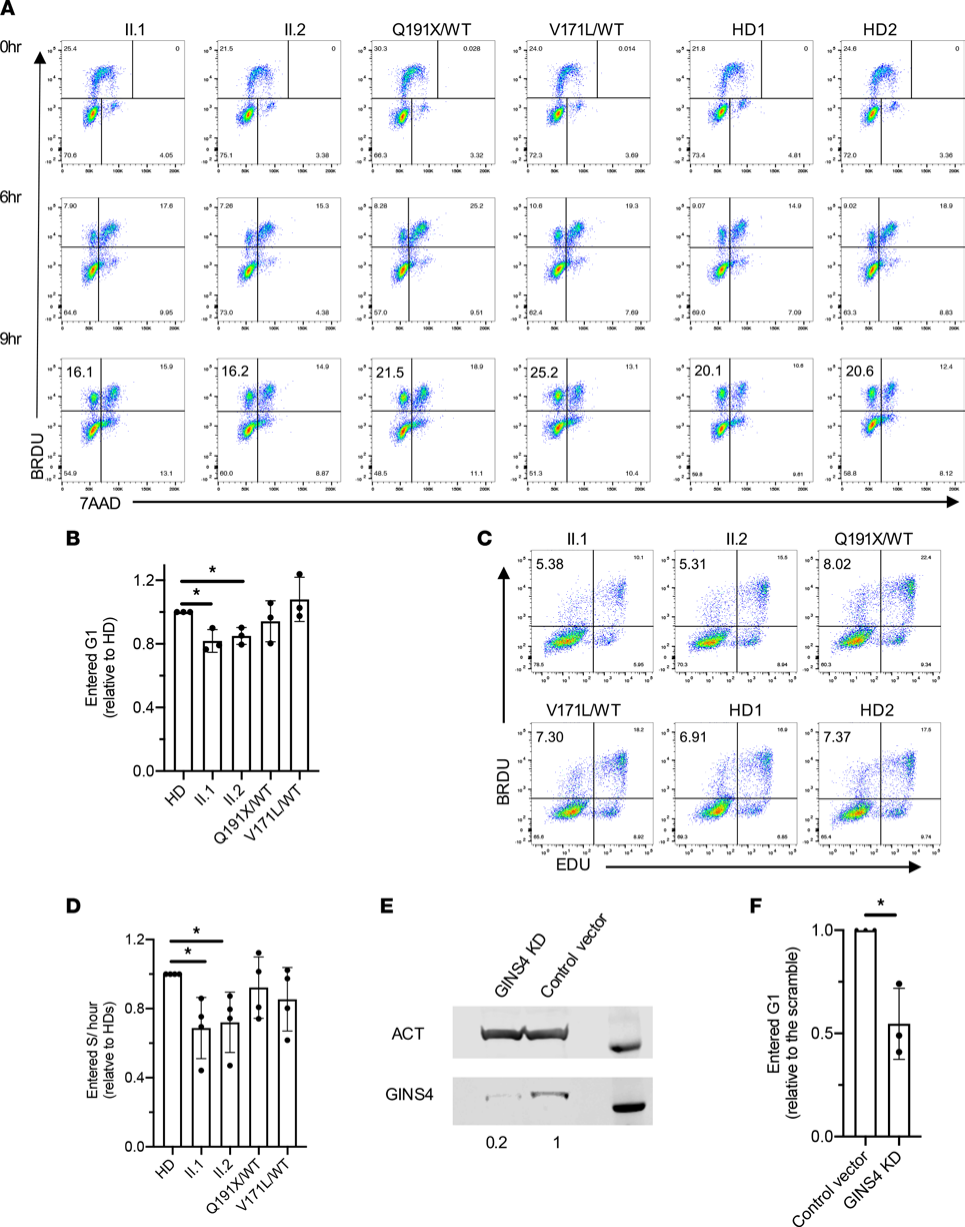
Individual-derived BLCLs and *GINS4*-KD RPE hTERT cells have a mild delay in cell cycle progression. (**A**) Cell cycle progression analysis in GINS4 family members and 2 HD-derived BLCL at early passages, shortly pulsed with BrdU and analyzed by FACS after 6 and 9 hours. One representative experiment of 3 independent replicates is shown. (**B**) Relative frequency of 2N BrdU^+^cells shortly pulsed with BrdU and analyzed by FACS after 9 hours in family members’ BLCL compared with pooled HD showing the mean ± SD of 3 independent experiments. *P* ≤ 0.05 1-sample 2-tailed *t* test and Wilcoxon test. (**C**) Representative plots of EdU BrdU double pulse of siblings, healthy parents, and HD-derived BLCL. (**D**) Relative frequency of EdU^−^BrdU^+^ cells compared with pooled HD values showing the mean ± SD of 4 independent experiments. *P* ≤ 0.05 1-sample 2-tailed *t* test and Wilcoxon test. (**E**) Representative GINS4 expression in *GINS4* KD and control vector RPE hTERT cell line by Western blot. Actin (ACT) is used as a loading control. (**F**) Relative frequency of 2N BrdU^+^ cells in GINS4 KD compared with the control vector displaying the mean ± SD of 3 independent experiments. *P* ≤ 0.05 1-sample 2-tailed *t* test and Wilcoxon test.

We first investigated the consequences of these variants on the cell cycle by treating the individual-derived cells with a short pulse of BrdU to measure the frequencies of cells in G0/G1, S, and G2/M phases. Interestingly, despite significantly lower GINS protein expression (~17%) and decreased assembly of the GINS complex, cell cycle analysis demonstrated that proband- and sibling-derived cells did not show marked aberrations of cell cycle phases (Figure 4A).

Given that the cell cycle was not grossly perturbed, we focused on proliferation dynamics by tracking the cell cycle progression of cultured cells over time. The proportion of cells arising from mitotic division was identified through BrdU labeling of DNA-synthesizing cells and the measurement of BrdU^+^ cells in G1 phase (diploid) 6 and 9 hours after the pulse. We found that cells from the individuals with compound heterozygous *GINS4* variants consistently had ~20% fewer cells reentering the G1 phase 9 hours after the BrdU pulse when compared with the BLCLs from HD and both parents (Figure 4, A and B). Next, we identified cells entering S phase through a double pulse labeling technique using 2 analogs of thymidine: EdU and BrdU. We sequentially pulsed the cells first with EdU and then with BrdU. The cells in S phase at the EdU pulse and exited S phase during the BrdU pulse became EdU^+^, while cells in S phase during both pulses became EdU and BrdU double-positive (EdU^+^BrdU^+^). Cells that entered S phase during the BrdU pulse were EdU^−^BrdU^+^. In the affected individuals (II.1 and II.2), we observed 5% of cells entering the S phase of the cell cycle, compared with 7% in HDs (Figure 4, C and D).

To advance this hypothesis and to establish a potential causal relationship between damaging *GINS4* variants and the observed cellular phenotype, we generated a *GINS4*-KD hTERT–immortalized retinal pigmented epithelial (RPE human telomerase reverse transcriptase [hTERT]) cell line that expresses levels of GINS4 similar to those observed in the proband and sibling-derived cells without affecting the expression of other *GINS* genes (Figure 4E and Supplemental Figure 5A), and we analyzed the proportion of cells arising from mitotic division. We found that *GINS4*-KD RPE hTERT cells consistently had a ~40% decrease in cells arising from mitosis (Figure 4F and Supplemental Figure 5B). Thus, the presence of physiological levels of functional GINS4 appears to be required for mitotic exit, which was significantly and reproducibly defective to a mild extent in affected individuals’ cells.

### GINS4 variants do not cause major DNA replication fork instability or increased DNA damage.

**Figure 5:**
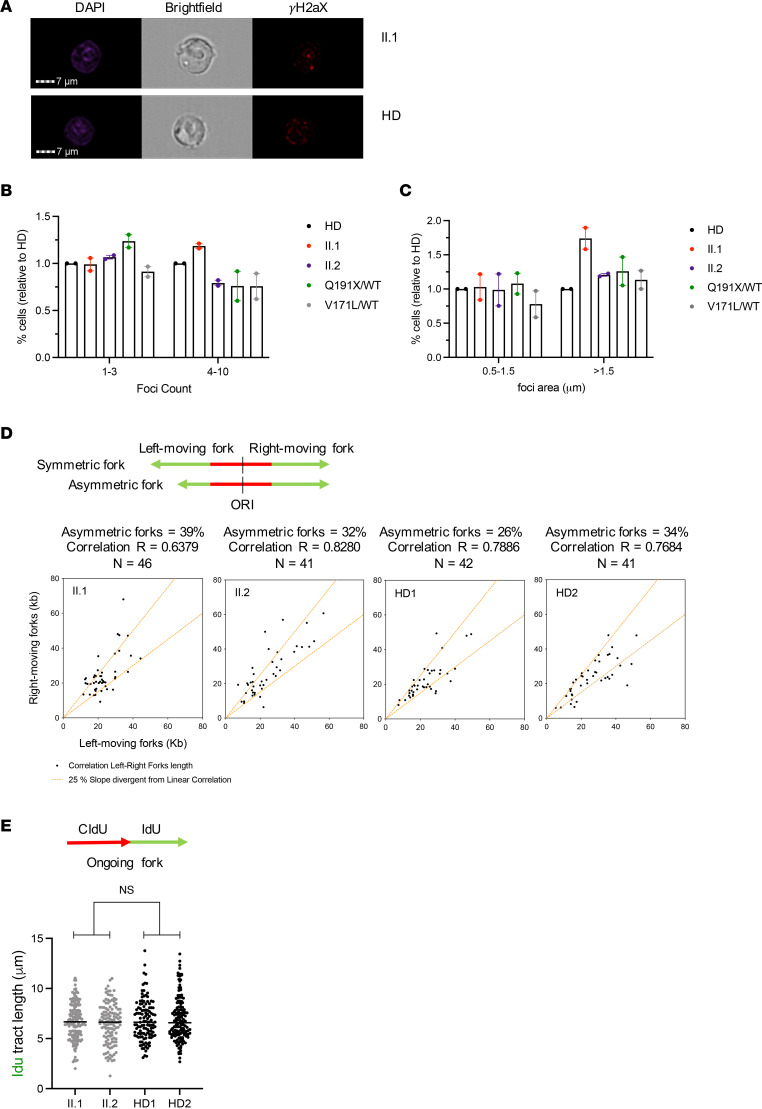
Individual-derived BLCLs do not show increased DNA damage or replication fork aberrations. (**A**) Representative imaging flow cytometry images of individual II.1 and HD; DAPI (left), bright-field (center), and γH2aX (right) are shown. (**B**) Relative percentage of individuals’ cells with low foci count (1–3 foci) and high foci count (4–10 foci) compared with HD. Data show the mean ± range of 2 independent experiments. For each experiment, 5,000 cells were analyzed. (**C**) Relative percentage of individuals’ cells with low foci area (0.5–1.5 μm) and high foci area (>1.5 µm) compared with 1 HD. Data show mean ± range of 2 independent experiments. For each experiment, 5,000 cells were analyzed. (**D**) Analysis of fork symmetry in the indicated individual-derived BLCLs. A schematic representation of symmetric and asymmetric forks is shown. The graph shows the length of the left fork (*x* axis) plotted against the length of the right fork (*y* axis) for each replication origin (indicated with dot). The fork was considered asymmetric if the ratio between the left-fork length and the right-fork length deviated by more than 25% from 1. *N* represents the number of forks analyzed in 2 independent biological replicates. *R* represents the linear correlation coefficient. (**E**) Analysis of fork speed in the indicated individual-derived BLCLs. Dot plot graphs with the median of IdU tract lengths (μm) are shown and individual points are derived from 2 independent experiments. *P* ≥ 0.05 by Mann-Whitney *U* test.

To investigate whether the delayed progression from mitotic division was a consequence of RS, we evaluated the number and area of γH2AX foci, a marker of DSB, in proband- and sibling-derived BLCLs by imaging flow cytometry. In the proband, we found only a slight increase in the frequency of cells with a higher number (4–10 foci) and area (>1.5 μm) of foci (Figure 5, A–C). We did not observe an increase in DNA damage in the proband’s sister, relative to her parents or HD. To further evaluate the response to RS, we looked at DNA damage tolerance activation and cell cycle arrest checkpoints. Posttranslational modifications of PCNA, mainly through monoubiquitination at lysine 164 (ubPCNA), which occurs specifically at active forks in response to RS, functions critically in DNA damage tolerance and promotes translesion DNA synthesis (41). Phosphorylation of CHK1 (pCHK1) at serine 317 by ataxia-telangiectasia and rad3-related (ATR) on damaged chromatin results in CDC25A degradation and cell cycle arrest or delay (42). We, therefore, assessed ubPCNA and pCHK1, both at baseline and following 0.5 and 2 mM hydroxyurea treatment, by Western blot and found no significant differences in the protein levels in either the proband or his sister, relative to their parents or the HD (Supplemental Figure 6).

To more deeply assess the function of GINS in the elongation of the replication forks, we evaluated the symmetry and speed of forks in the BLCL using DNA fiber analysis. Consistent with the absence of increased DNA damage, we found that the fork symmetry (Figure 5D) and fork speed (Figure 5E) were comparable in the proband and his sister when compared with the 2 HDs. Thus, the proband- and sibling-derived BLCLs demonstrated mild impairments in mitotic exit and entry into S phase, as well as mildly increased DNA damage in the proband, without detectable abnormalities of replication fork speed and symmetry.

### Impact of GINS4 variants on gene expression profiles in NK cells.

To further characterize NK cell development and gain insights into the presence of *GINS4* variants, we assessed the expression of immune- and cell cycle–related genes by NanoString gene expression analysis (Customized Human Immunology v2 gene set, Supplemental Table 3). Analyses were performed on freshly isolated NK cells from individual II.2, her father (I.2) and 3 HDs as controls. Given the near absence of NK cells in the peripheral blood of individual II.1, we could not isolate sufficient numbers of NK cells from him to include in our analyses. However, the presence of both of the damaging *GINS4* variants in individual II.2, along with a similar NK cell phenotype, allowed for estimation of any impact.

**Figure 6:**
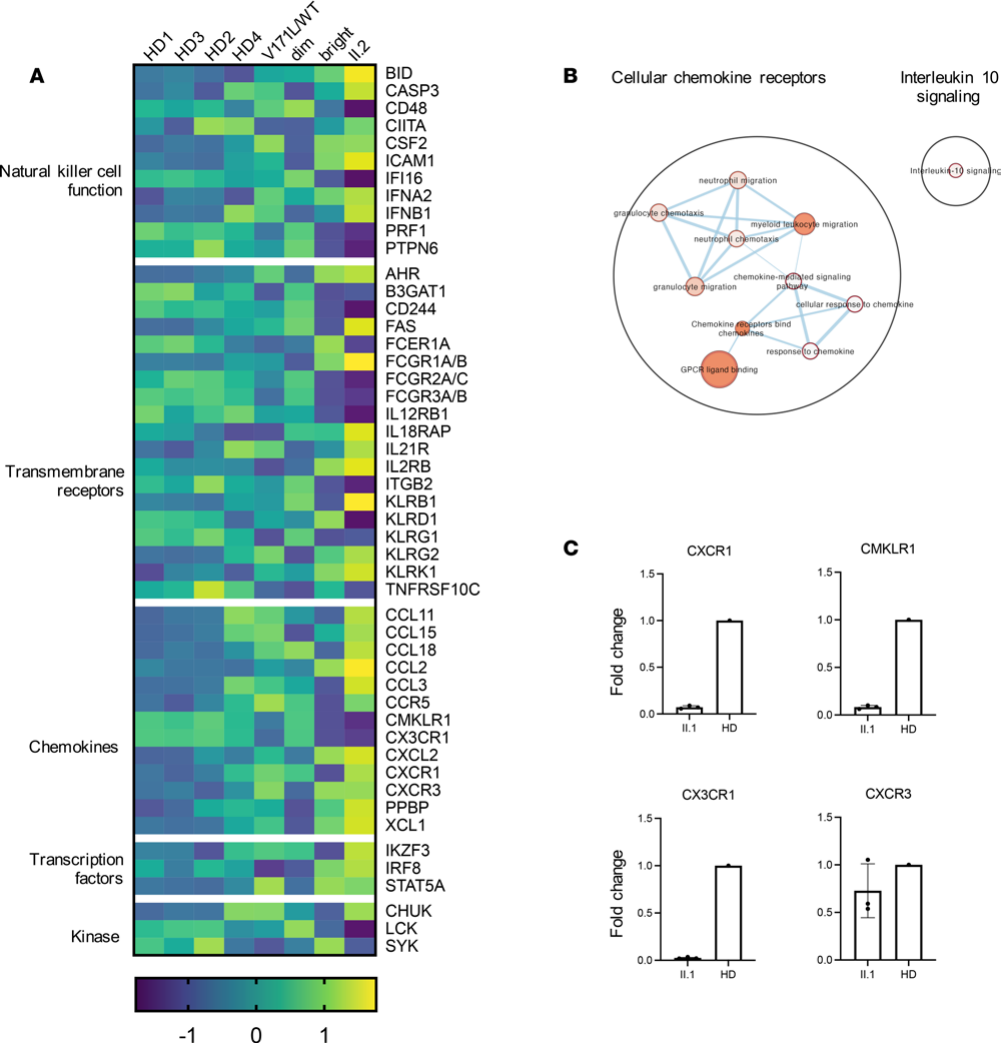
Immune profile of enriched primary NK cells demonstrates less mature NK cell phenotype. (**A**) Heatmap of significantly deregulated expression of genes relevant to NK cell biology from enriched NK cells from individual II.2, her father (I.2), and 4 healthy donors, with sorted CD56^bright^ and CD56^dim^ NK cells from a single healthy donor also shown for reference. (**B**) Visualization of the pathway enrichment analysis performed with g:Profiler and visualized with Cytoscape. (**C**) Confirmatory qPCR analysis of CXCR1, CMKLR1, CX3CR1, and CXCR3 in proband II.1 compared with control.

NanoString analyses identified 209 genes with a greater than 2.5-fold up- or downregulation in II.2 relative to all 3 HDs (Supplemental Table 4). These included 35 genes of particular relevance to cytotoxicity, chemotaxis, and proliferation of NK cells (Figure 6A), namely the effector molecules and cell surface receptors *PRF1*, *CD244*, *FCGR3A/B*, *KLRG1*, *B3GAT1*, *IFNB1*, and *AHR*, and transcription factors with known roles in NK cell maturation *IKZF3*, *STAT5A*, and *IRF8*. Pathway enrichment analysis of these 209 differentially expressed genes revealed “response to chemokine receptors” as the most statistically significant pathway (Figure 6B and Supplemental Table 5). In keeping with the mild defects in cell cycle progression, no enrichment in cell cycle–related genes was defined. Pathway enrichment analysis of the proband’s father (I.2) relative to HDs did not show any significantly altered pathways, despite the heatmap showing an intermediate expression profile, which may reflect the heterozygous state of the *GINS4* truncation variant (Figure 6A). Among the chemokine receptor genes, *CX3CR1* and *CMKLR1* appear the most downregulated, while *CCR5*, *CXCR1*, and *CXCR3* were upregulated (Figure 6A and Supplemental Table 4).

Given the relative overrepresentation of the CD56^bright^ subset within the NK cell population of individual II.2, we sought to determine whether the changes in gene expression reflected a greater proportion of CD56^bright^ NK cells in the enriched NK cell population. For this purpose, we compared the gene expression profile of the purified NK cells from individual II.2 to purified CD56^dim^ and CD56^bright^ NK populations from HD PBMC. The gene expression profile of II.2 was most similar to that of CD56^bright^ cells, especially when considering transmembrane and chemokine receptors (Figure 6A). NK cell subsets display different chemokine receptor repertoires that reflect differential functional and localization properties, and CD56^dim^ preferentially express CXCR1, CX3CR1, and CMKLR1 and localize to peripheral tissues, while CD56^bright^ NK cells express CCR5, CCR7, and higher levels of CXCR3 and CXCR4; furthermore, they localize to secondary lymphoid organs (43). In addition, *PRF1* (perforin), *FCGR3A* (CD16), *B3GAT1* (key enzyme in CD57 biosynthesis), and *KLRG1*, which are highly expressed within the CD56^dim^ subset, were underrepresented in the NK cells of individual II.2.

To validate the selected differentially expressed genes identified in our analyses, we performed confirmatory quantitative PCR (qPCR) on isolated NK cells from individual II.1 and 1 HD and found decreased expression of *CX3CR1*, *CMKLR1*, and *CXCR1*, as well as normal *CXCR3* expression in II.1 compared with control (Figure 6C). In addition, we evaluated these chemokine receptors by flow cytometry analysis of PBMC, resting or stimulated with IL-15 for 48 hours. We confirmed lower expression of CX3CR1 and higher expression of CXCR3 in individual II.2 in total NK cells at baseline. Individual II.1 showed normal or lower MFI of CXCR3. Notably, increased expression of these receptors after IL-15 stimulation was not found in the individuals with *GINS4* variants, especially the CXCR1 receptor (Supplemental Figure 7). Thus, altered expression of chemokine receptors in NK cells may underly causative effects of *GINS4* mutations, as suggested by the altered gene expression found in II.2. Additionally, differences in gene expression reflect an increased representation of the CD56^bright^ subset, suggesting that it was likely at least in part a consequence of impaired NK cell maturation.

### NK cell differentiation requires full expression of GINS4.

**Figure 7:**
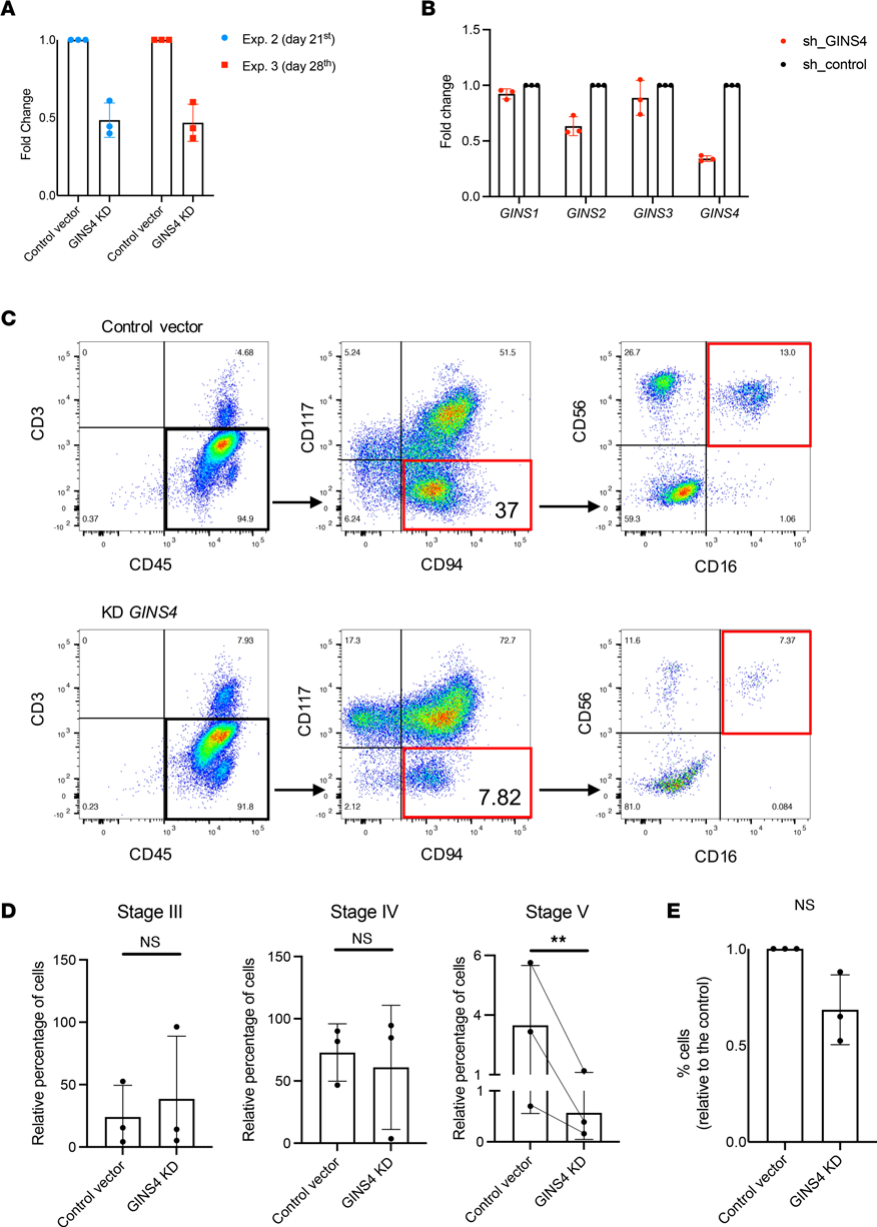
*GINS4*-KD in CD34^+^ hematopoietic precursors impairs NK cell maturation. (**A**) *GINS4* expression measured by qPCR in differentiating cells at day 21 and 28 of differentiation showing technical triplicates ± SD from the 2nd and 3rd experiments, respectively. (**B**) Evaluation of off-target effect on other *GINS* by qPCR analysis of *GINS4*-KD differentiating CD34^+^. (**C**) Representative flow plots of NK cell terminal differentiation of 3 independent experiments. (**D**) Relative frequency of CD45^+^CD3^−^ differentiating NK cells in each maturation stage as previously described (Supplemental Figure 8). Data show mean ± SD of 3 independent experiments. *P* ≤ 0.05 by paired 2-tailed *t* test. (**E**) Percentage of CD45^+^CD3^−^ cells in *GINS4*-KD condition compared with the control. Data shown are mean ± SD from 3 independent experiments.

Flow cytometry of PBMC from individuals II.1 and II.2 in concert with NanoString analysis demonstrated a reduced frequency of NK cells with increased CD56^bright^ immature cells relative to the CD56^dim^ subset. CD56^bright^ NK cells are thought to be precursors of the CD56^dim^ subset, and previous studies suggest that proliferation is associated with terminal maturation of the CD56^bright^ subset (44). To evaluate an intrinsic requirement for GINS4 during NK cell differentiation, we introduced the shRNA against *GINS4* that was used to generate the *GINS4*-KD RPE hTERT cell line (Figure 4E and Supplemental Figure 5A), by lentiviral transduction into CD34^+^ hematopoietic precursor cells (HPC) from a HD to generate *GINS4*-KD HPCs that were used for the generation of mature NK cells by in vitro differentiation (Supplemental Figure 8A) (19, 45). *GINS4* expression was monitored during the differentiation process to confirm a reduction in mRNA expression (Figure 7A). Furthermore, we assessed the off-target effects on the expression of other *GINS* members by performing qPCR 10 days after differentiation, and we found only mild off-target effect on *GINS2* expression (Figure 7B). The mechanism by which this downregulation happens is unknown but may be related to the decreased expression of other GINS proteins shown in Figure 3, A–C, and previously reported by others (14, 37). Nevertheless, we cannot exclude the contribution of some off-target effect on *GINS2* in the CD34^+^ context.

Following 32 days of culture, NK cell developmental stages were assessed by flow cytometry using previously described phenotypic markers to track the progression from stages 1 to 5 (Supplemental Figure 8, B and C) (46, 47). We found slight and inconsistent differences in stages 3 and 4 cell frequencies and significantly decreased frequency of fully mature NK cells in stage 5 when comparing control and knockdown conditions (Figure 7, C and D). No cells in stages 1 or 2 were found. Although the limited number of cells prevented us from performing cell cycle analysis, we observed the number of CD45^+^CD3^–^ cells derived from CD34^+^ HPC with *GINS4* shRNA and compared them with the nonspecific controls. A decreased number of cells was found in the *GINS4*-KD (albeit not statistically significant), suggesting at least some impact on proliferation relative to the effects on NK cell development (Figure 7E). Collectively, these data demonstrate that decreased expression of GINS4 during in vitro differentiation recapitulates the NK cell phenotype observed in the peripheral blood of individuals with compound heterozygous-damaging *GINS4* variants. Importantly, it suggests that NK cell maturation requires physiologic GINS4, with this requirement detected most notably at the terminal stages and in an NK cell–intrinsic manner.

## Discussion

Here, we describe a familial case of primary immunodeficiency, characterized by a reduced frequency of NK cells, impaired NK cell function, and neutropenia caused by compound heterozygous mutations in *GINS4* — namely, a stop codon and a missense variant. Biochemical assays demonstrated decreased expression and interaction capabilities of both variant alleles with GINS1 and GINS3 proteins. In addition, the stop codon allele was detectable in protein lysate, suggesting that it is expressed and partially escapes NMD.

Immunological phenotypes of the 2 individuals with both *GINS4* variants include a substantial reduction of circulating NK cells (with relatively increased frequency of CD56^bright^ NK cells) and neutropenia; however, they have normal B and T cell populations. The proband presented clinical features outside of immunodeficiency, including growth retardation and intrauterine growth restriction. The proband also had more substantive clinical immunodeficiency from an infectious standpoint. While the mechanism by which disease severity is increased in the individual II.1 (older sibling) is unknown, it may be related to an early CMV infection that was diagnosed at 11 months of age, representing some degree of a critical infection at a critical time. The proband’s younger sister (II.2) also had a fixed immunological phenotype, with defective NK cells and neutropenia. She, however, did not experience severe or unusual infections that could be ascribed to a deficiency of NK cells, perhaps with the exception of recurrent herpes simplex. NK cells are an essential and nonredundant component of the human immune system in controlling diseases, with established roles in herpes viral infections and tumors, as highlighted through the study of individuals with NKD and other conditions (48, 49). Experience to date with individuals having NKD demonstrates that clinical presentation may occur at young ages but can also appear much later in life. In our program for NKD investigation, the initial presentation for clinical immunodeficiency is most commonly found in individuals older than 8 years (50). While her clinical history prevents us from considering the proband’s sister as having clinical immunodeficiency, her susceptibility to symptomatic herpes viral infection should be taken into account.

Both parents seem clinically unaffected, but the mother, harboring the Q191X allele, shows some signs of impaired NK cell function measured by in vitro assays, possibly as a result of the deleterious effects of the truncated protein that escapes NMD and interferes with GINS complex formation. Limited availability of biological samples prevented further extensive analysis of the NK cell phenotype and function from the parental carriers of the single variants. The impaired NK cell function in the mother with the Q191X allele in the context of seemingly normal NK cell numbers and phenotype suggests that this variant, which is less deleterious alone than as a compound heterozygous mutation, highlights a potential requirement for GINS4 directly in NK function. However, in the absence of in-depth phenotyping, maturational analyses, and cell biological studies, the mechanism by which the mother’s NK cell function is affected remains unclear. Despite this observation, immune transcriptome analysis of circulating NK cells from the proband’s sister (II.2) identified a genetic profile showing that these cells align closely with the CD56^bright^ NK cell subset. Also, compared with the analysis of her father’s NK cells, carrying the V171L mutation (I.2), the changes in gene expression that we found further suggest that incomplete NK cell maturation may be associated with the Q191X *GINS4* mutation that leads to accompanying impaired cytotoxic function. No genes associated with apoptosis or cell survival were dysregulated, suggesting that a specific defect in development impacts both the proband and his sister’s NK cells.

In addition to the NK cell defect, the proband and his sister demonstrated neutropenia, which has been described in GINS1 deficiency (14), but not in MCM deficiencies. While the mechanism underlying neutropenia and NKD in GINS is unknown, neutrophils and NK cells are known to regulate each other’s function, survival, and development (51). In mice lacking neutrophils due to mutations in *Gfi-1*, NK cells hyperproliferate, yet they have poor survival, impaired function, and a block in maturation (52). Furthermore, some individuals with congenital neutropenia have a decreased frequency of CD56^dim^ NK cells in the periphery and NK cell functional impairment (52, 53), a phenotype very similar to that seen in MCM- and GINS-deficient individuals. In the proband (II.1) described in our study, as in GINS1 deficiency, neutropenia was corrected by treatment with G-CSF. Since G-CSF does not correct the NKD in our proband or GINS1-deficient individuals, we hypothesized that GINS4 is intrinsically required for NK cell development. In support of this, when we modeled *GINS4* partial depletion in CD34^+^ precursors using shRNA and NK cell in vitro differentiation, the NK phenotype observed in the proband, including impaired terminal differentiation and lower but not significant impairments in cell proliferation, was recapitulated. Thus, we propose that GINS4 is specifically required for the development of NK cells in a cell-intrinsic manner. While we do not provide any direct parallel evaluation of neutrophils, these are short-lived cells (8–20 hours), and exposure to NK-derived cytokines IFN-γ and GM-CSF prolongs their survival (54). Furthermore, activated NK cells can influence neutrophil migration through the NK cell–derived CC-cytokine secretion. Our gene expression analysis showed significant differences in CC-chemokines, such as CCL3, and it is unclear if the NKD may, therefore, impact neutrophils in GINS4- and GINS1-deficient individuals. In other words, perhaps the neutrophil deficiency is extrinsic and secondary to the deficiency of NK cells. Alternatively, there may be a unique requirement associated with immune cell proliferation and apoptosis that is common to NK cells and neutrophils (53).

It is striking that only 8 NKD have been described (49). Among them, 4 are caused by mutations in the origin of replication firing genes, *MCM4*, *GINS1*, and *GINS4*, which are integral components of replicative helicase, and in *MCM10*. Moreover, mutations in other DNA replication–associated genes that lead to NK cell abnormalities highlight the importance of the replicative helicase complex and, more broadly, of DNA replication in NK cell biology. For instance, pathogenic variants in DNA polymerase subunits (polδ, polε, and polα) have also been linked to immunodeficiency, with variable phenotypes including NK, T, and B cell deficiency and development-related features (55–58). Furthermore, a similar immature phenotype of NK cells, with increased CD56^bright^ CD16^−^ frequency, has been described in individuals harboring hypomorphic recombinase-activating gene (RAG) mutations (59). While NK cells, as innate immune cells, develop independently of TCR and BCR signaling, mature NK cells subsets that experience RAG-mediated double-stranded breaks during development inherit cellular fitness to overcome DNA RS (60). Therefore, RAG likely mediates a role or function beyond VDJ recombination, and the fact that B and T cells experience RAG-mediated DSB during development suggests that DNA damage repair pathways can be differently regulated. This implies that NK cells may utilize pathways related to DNA damage repair and replicative fitness that are unique from T or B cells, uncovering a unique sensitivity to variants that affect DNA replication. In other words, NK cell maturation and development may be specifically intolerant to negative pressure on DNA replicative function.

The key immunological feature of individuals with helicase deficiency is the decreased frequency of NK cells. Although CMG proteins are ubiquitously expressed, these individuals all have a conserved NK cell phenotype with excess CD56^bright^ populations and, in addition, may have other milder non–NK cell immune defects. For instance, there is a decreased frequency of MAIT, invariant NKT, and circulating innate lymphoid cell (ILC) precursors in *GINS1* cases, suggesting that the effect of *GINS1* deficiency may be slightly broader than those of *MCM4* or *MCM10* deficiency, which were seemingly restricted to the NK cell lineage. In addition, as *mcm4-*, *mcm10-*, and *gins4*-null mutations are lethal in mice (61–63) and no homozygous LoF cases of any are present in gnomAD database, the specific variants associated with NKD are clearly only partial in their aberration of function.

MCM4-deficient PBMCs demonstrated a partial impairment of lymphoid lineage survival and increased apoptosis upon IL-2 and IL-15 stimulation that is most pronounced in NK and NKT cells than in conventional T cells and B cells (15). Since NK cells proliferate 3–4 times faster than T cells (44), changes in cell cycle may lead to different regulation of DNA synthesis, including the usage of origin of replication and fork speed maintenance. Our in vitro model found impaired terminal maturation and lower frequencies of differentiated NK cells, suggesting a decreased proliferation capacity of *GINS4*-deficient precursors. Along these lines, we observed less activation and maturation after IL-15 treatment in vitro, without evidence of survival impairments. Despite the damaging nature of *GINS4* variants, the proband- and sibling-derived BLCLs and the *GINS4*-KD RPE-hTERT cell lines had a mild cell cycle–delay phenotype without DNA replication–associated impairments or DNA damage, besides a slight increase in the γH2AX signal detectable only in the proband.

DNA replication is tightly regulated, and different cell lineages appear to have unique requirements for CMG concentrations. Neutrophil precursors divide efficiently to supply the daily number of neutrophils needed; therefore, they are likely severely affected by DNA replication defects. However, NK cells are a longer-lived population and are not expected to divide so extensively. Furthermore, changes in the expression and function of helicase proteins mark differentiation from the earliest immune cell precursors. HSC primarily reside in G0 and have distinct metabolic and cellular responses to DNA damage characterized by high-fidelity repair mechanisms to maintain a high standard of genomic integrity (64). As HSCs progress through differentiation, cell divisions become more frequent and rapid, requiring rapid loading of MCM complexes; lower levels of MCM proteins result in limited hematopoietic functionality and increased DNA damage associated with RS (65). In addition to limiting proliferation capacity, persistent γH2AX foci block the transcription of damaged DNA, further preventing cell differentiation (66). Defective DNA damage tolerance, which enables bypassing fork stalling to prevent RS, leads to a reduced number of HSCs and skewed differentiation toward myeloid/erythroid lineages to safeguard oxygen supply at the expense of the number of common lymphoid progenitors (CLP) that give rise to T, B, and NK cells (67). Therefore, while impairment in NK cell maturation in helicase NKD is detected at the stage of terminal differentiation, it is conceivable that the earliest hematopoietic precursors are the ones actually affected, leading to changes in downstream lineage decisions.

The variable phenotypic outcomes associated with DNA replication genes and in vitro functional studies of primary cells may also lead to additional consideration regarding the roles of these proteins. For instance, the *GINS1* mutations, retaining residual activity of 16%, cause impaired checkpoint activation and a slight increase in DNA damage, with normal replication fork progression (14). Our results, showing a cell cycle delay, suggest that a mild mitotic exit block occurs independently of DNA damage, consistent with previous reports demonstrating a noncanonical role for the GINS4 subunit in maintaining the centrosome structure during mitosis (40). Alternatively, the proliferation delay may result from slower DNA replication of highly repetitive regions like centromeres and telomeres. It is possible that more damaging *GINS4* mutations could cause DNA replication impairments and DNA damage accumulation through the genome, as has been described when GINS4 expression is < 10% (39). In addition, beyond unwinding dsDNA during the replication, the CMG helicase can modulate the formation of RNA-DNA hybrids generated by the transcription, which may accumulate and interfere with the DNA replication apparatus during cell development (68).

Thus, full origin licensing and noncanonical roles of CMG may both be important for functional NK cells. Further experiments are needed to understand if increased and persistent DNA damage due to RS can activate apoptosis or cell cycle arrest to prevent NK cell maturation. Alternatively, as hematopoietic precursors differentiate and undergo complex transcriptional programs with chromatin remodeling, the interplay between transcription and replication, fork speed imbalance, impairments of the full firing of early, and late origins and reduced unwinding capability may lead to altered transcription patterns that result in impaired NK cell maturation. Finally, the dysregulation of chemokine receptors could impact NK cell homeostasis and maturation. Lymphocyte precursors that give rise to NK cells circulate in the blood and migrate through multiple lymphoid tissues, and terminal maturation is associated with the acquisition of complex and heterogeneous migratory behaviors (69). Still, the lack of expression of migration-associated genes in the presence of *GINS4* mutations in our gene expression experiments suggests that it is unlikely that defective homing or trafficking of NK cell precursors further impacts NK cell maturation and homeostasis in this context.

## Methods

### Cell isolation and cell lines.

PBMC were isolated from whole blood by density gradient centrifugation using Ficoll-Paque. CD34^+^ cells for in vitro differentiation were purchased from StemCell or isolated from discarded apheresis product from routine red cell exchange at Columbia University Medical Center. Primary B lymphocytes were immortalized by EBV infection (70) and maintained in RPMI supplemented with 10% FBS (Atlanta Biologicals, catalog S11150), penicillin-streptomycin (PenStrep; Thermo Fisher Scientific, catalog 15140), L-glutamine, nonessential amino acid (NEAA), sodium pyruvate, and HEPES. HEK293T fibroblast lines were maintained in DMEM supplemented with 10% FBS, 1% PenStrep, and L-glutamine. Immortalized retinal pigmented epithelial cells (hTERT-RPE1) were purchased from ATCC and maintained in DMEM:F12 supplemented with 10% FBS and 1% PenStrep. K562 cells are maintained in RPMI medium supplemented with 10% FBS, 1% PenStrep, and 1% GlutaMAX at 37°C, and 5% CO_2_. All cell lines were confirmed negative for mycoplasma.

### Immunophenotyping by flow cytometry.

Immunophenotyping of individuals’ PBMCs, isolated from fresh blood or frozen samples, was performed using multicolor flow cytometry analysis. Fluorophore-conjugated antibodies used for individuals’ samples are listed in Supplemental Table 6. Cells were washed in PBS with 2% FBS, incubated with the antibodies and viability dye at room temperature, protected from light for 30 minutes, and washed and resuspended in 500 μL of PBS with 2% FBS. To evaluate CD107a, perforin, and IFN-γ expression in NK cells, PBMC were stimulated with 1 μg/mL of PMA and 1 μg/mL of Ionomycin for 4 hours. Brefeldin (BD Biosciences), Golgi stop (BD Biosciences), and anti-CD107a were added. Cells were stained and permeabilized with BD Cytofix/Cytoperm kit (BD Biosciences).

To evaluate the maturation, activation, and chemokine receptors of NK cells, PBMCs were thawed and stimulated with 15 ng/mL of IL-15 for 48 hours. After incubation, cells were recovered, stained, and permeabilized with BD Cytofix/Cytoperm kit (BD Biosciences). Unstimulated cells were added as a control, and fluorescence minus one (FMO) controls were included for each marker. Cells were acquired on BD Fortessa configured for 14 colors or on Cytoflex Flow cytometer and analyzed in Flow Jo v.10.8.1.

### NK cell cytotoxicity assays.

^51^Cr release assays were performed to evaluate the cytotoxicity of individuals’ NK cells using PBMC against K562 erythroleukemia as previously described (71). LU were defined as the number of effectors required to lyse 10% of targets cells. For PBMCs, LU were calculated as the inverse of 1 million cells using 0.01 million targets per well. For NK cells, LU were subsequently normalized by multiplying by the percent of NK cells in the PBMCs sample.
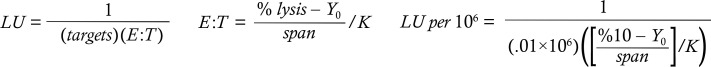


### Whole exome sequencing and analysis.

Individuals’ genomic DNA was used to perform whole-exome sequencing. As previously described (72), exonic portions of DNA were enriched with custom VCrome probes (NimbleGen) and sequenced using Illumina HiSeq 2500 equipment. Variants with an allelic frequency of less or equal to 0.01 in the BHCMG, ESP5400, and 1000 Genomes databases (https://mendeliangenomics.org, https://evs.gs.washington.edu/EVS/, http://www.1000genomes.org) were bioinformatically selected as previously described (72). Prioritized variants were confirmed by Sanger sequencing of PCR product. Primers were designed using Primer Blast. 4Peaks software (Nucleobytes) was used to visualize sequence trace files. Multiple Sequence Alignment shown in Figure 2D was executed using the online tools freely available at https://www.ebi.ac.uk/Tools/msa/

### Plasmids, transfection, and transduction.

N-terminal MYC-tagged *GINS4* coding sequence (NM_032336) was cloned into the pDONR201 plasmid. Site-directed mutagenesis reactions to generate missense (V171L) and stop codon (Q191X) were performed by Epoch Life Science. In total, 2 μg of WT, V171L, or Q191X *GINS4*–expressing plasmids were transfected in 3 × 10^5^ HEK293T cells using Lipofectamine 3000 according to manufacturer instructions (Invitrogen). Co-IP experiments were performed 48 hours after transfection.

Stable *GINS4*-KD cells were generated by transducing RPE-hTERT or CD34^+^ precursors with pGFP-C-shLenti (catalog TL304343) or control vector (catalog TR30021) purchased from Origene. Polybrene or Transdux Max reagent (LV850A-1) was used for transduction according to the manufacturer’s instructions (Systems Bio). The most effective short hairpin (shB) vector against *GINS4* was prior tested by HEK293T transduction. CD34^+^ precursors or RPE-hTERT transduced cells were isolated by FACS for GFP^+^ signal 3 days after transfection. In RPE-hTERT, the GINS4-KD efficiency was measured by Western blot. In NK differentiating cells from HPCs, *GINS4* expression was measured by qPCR as described below.

### Western blotting and co-IP.

BLCLs from family members or HDs, as well as KD RPE-hTERT or control RPE-hTERT, were lysed in RIPA buffer supplemented with 1× Halt Protease and Phosphatase Inhibitor cocktail (Thermo Fisher Scientific). For co-IP experiments, HEK293T cells transiently transfected with p-Lenti myc-*GINS4* plasmids and lysed 48 hours after transfection in Co-IP buffer (10 mM Tris-HCl [pH 7.5], 150 mM NaCl, 0.5M EDTA, 0.5% NP40, 1× halt proteinase phosphatase inhibitor) supplemented with 75 U/mL DNase I and 2.5 mM MgCl_2_ for 30 minutes at 4°C. Lysates were incubated with myc-Trap antibody coupled to magnetic agarose beads (ytma-20, Chromotek) or blocked with magnetic agarose beads as a binding control (bmab-20, Chromotek). Lysates or Co-IP eluates were separated by electrophoresis using a 4%–12% gradient gel and subsequently transferred to a nitrocellulose membrane. To saturate protein-binding sites, the membranes were incubated with 10% nonfat dry milk solution then incubated with the antibodies listed in Supplemental Table 6. Full uncut gels are available in the published online Supplemental Material.

### Cell cycle analyses.

We performed cell cycle analyses using the BrdU assay following kit instructions (BD Biosciences). Briefly, RPE-hTERT cells or BLCLs at early passages were incubated with 10 mM BrdU for 30 minutes, followed by fixation, incubation with anti-BrdU (559619, BD Biosciences), and staining with 7-AAD. To determine the proportion of cells arising from mitosis and reentering G1, 5 × 10^5^ cells were incubated with 10 mM BrdU for 30 minutes and were then washed twice in PBS and replated in complete media for 3, 6, and 9 hours prior to fixation and staining with anti 7-AAD and BrdU (BD Bioscience). Cells that reentered G1 were indicated as diploid (2N) BrdU^+^. To determine the proportion of cells entering S phase, sequential pulses of dual thymidine analogs were applied. First, 5 × 10^5^ cells were incubated with 10 μM EdU for 2.5 hours; they were then washed twice in PBS and incubated with 10 mM BrdU for 2.5 hours. After the DNA denaturation step in the BrdU protocol (BD Biosciences), cells were click labeled to detect EdU and were then stained with the anti-BrdU antibody to detect BrdU using the Mobu-1 clone antibody (B35129, clone Mobu-1, Invitrogen), which does not have cross-reactivity to EdU. Cells that had entered the S phase were indicated as BrdU^+^EdU^–^. Samples were acquired by flow cytometry on a Bio-Rad ZE5 with a 24-color configuration and analyzed using FlowJo software. We quantified the frequency of cells found in cell cycle stages by gating strategy.

### DNA damage analysis.

Analysis of DNA damage foci was performed by imaging flow cytometry. Briefly, individuals’ and HDs’ BLCLs were fixed and permeabilized using FoxP3 transcription buffer (eBioscience). After washing, samples were incubated with anti-γH2AX (clone N1431 catalog 560447, Alexa Fluor 647, BD Bioscience) for 1 hour, followed by DAPI staining. For each experiment, at least 5,000 single, focused cells per sample were acquired on an ImageStream MkII (EMD Millipore) with a 60× objective and analyzed using IDEAS software (EMD Millipore). First, a compensation matrix, calculated using single-stained controls, was applied to all batched samples. In a preliminary analysis, a flow cytometry–based approach was used to gate single, focused, and live cells. Then, we selected a “training cell,” containing 3–4 well-defined spots, to create a combined mask designed to minimize the artifacts. It consists of a spot mask to identify the spots, a watershed mask to separate 2 close spots, a range mask to define the spot size and shape, and an intensity mask to set a background threshold. The combined mask was manually tested on multiple cells and then applied to all samples in a batched analysis. Finally, spot count and spot area features were calculated using the mask.

### DNA replication fibers analysis.

BLCLs were first incubated with 25 μM chlorodeoxyuridine (CldU) for 10 minutes, washed twice in warm PBS, and then incubated with 125 μM iododeoxyuridine (IdU) for 15 minutes. DNA fibers were prepared as previously described (73). Replication tracks images were acquired on a Nikon Eclipse 90i microscope fitted with a PL Apo 40×/0.95 numerical aperture (NA) objective. The length of each tract was measured manually using the segmented line tool on ImageJ software (NIH). In each experiment, 100 or more tracts were measured. Statistical differences in the distribution of DNA fiber tract length were determined by the Mann-Whitney *U* test. For fork symmetry analysis, the length of labeled tracks was converted into kilobases using the conversion factor 1 μm = 2.59 kb. We used Pearson correlation test to analyze the correlation between the length of left-moving and right-moving forks.

### RNA isolation and gene expression analysis.

Individuals’ NK cells were isolated from PBMC using RosetteSep negative selection (StemCell Technologies), followed by FACS to greater than 95% purity. We performed a gene expression analysis of a customized Human Immunology v2 probe set designed to include cell cycle genes (Supplemental Table 3). NanoString output was processed using NanoString nCounter. The mean of 8 negative control probes was used for background correction. The geometric mean of 15 housekeeping genes was used to normalize RNA content. Genes up- and downregulated greater than 2.5-fold in samples relative to the mean of all 3 HD controls were ranked and included in pathway analysis. Data were exported to g:Profiler (https://biit.cs.ut.ee/gprofiler_archive2/r1760_e93_eg40/web/index.cgi) for pathway analysis and visualized with Cytoscape as previously described (74).

### In vitro NK cell differentiation.

CD34^+^ hematopoietic precursors were isolated from discarded apheresis from Columbia University Medical Center or purchased from StemCell Technologies. CD34^+^ collection was performed using RosetteSep enrichment for initial lineage depletion and EasySep Human CD34 Positive Selection Kit II. CD34^+^ cells were cultured 1 day in serum-free Stem Span II media supplemented with 35 nM UM171 and 100 ng/mL of IL-6, thrombopoietin (TPO), Flt3L, and stem cell factor (SCF); they were then transduced as described above. GFP^+^ cells were sorted 3 days after transduction. In total, 2,000 cells were cocultured with irradiated EL08.1D2 stromal cells in the presence of IL-3 (during the first week only), IL-7, Flt3L, SCF, and IL-15 for 32 days. Then, cell surface receptors associated with progression from stage 1 to stage 5 of NK cell development were analyzed by FACS and quantified, as previously described (45).

### qPCR.

RNA was extracted using the RNeasy Micro kit (QIAGEN, catalog 74004), and first-strand cDNA was generated using superscript VILO MasterMix (Thermo Fisher Scientific, catalog 11756050). The qPCRs were performed in triplicate on a Roche LightCycler instrument using 25 ng of cDNA and TaqMan assays listed in Supplemental Table 7.

### Statistics.

Statistical analyses were performed with GraphPad Software (Prism 8.0). All data show mean ± SD, if not indicated otherwise. *P* value ≤ 0.05 was considered significant. Single comparisons were analyzed using 2-tailed Student’s *t* test, when data were normally distributed, or Mann-Whitney *U* test when data were not normally distributed. One-sample 2-tailed Student’s *t* test was used to compare the mean of an experimatal sample to the wild type normalized to 1.

### Study approval.

The informed consent for study purposes was signed by all participants. Baylor College of Medicine and Columbia University granted approval for the study (BCM IRB-H-29697 and H-30487; CUIMC IRB-AAAR7377).

## Author contributions

AT, GL, and AC analyzed and interpreted the DNA replication fibers experiments. IKC and JRL performed the genetic analysis. MCP and SAS produced the cell lines and the lentiviral vectors. MCP, ERJ, and NO acquired and analyzed the T cell and the extended NK cell panels provided in Supplemental Figures 1, 2, and 7. MIC designed, conducted, and analyzed all the other experiments appearing in the figures. JCAB and LVE collected and coordinated the clinical data. MIC, EMM, and JSO interpreted the data and wrote the manuscript. All the authors contributed to the manuscript.

## Supplementary Material

Supplemental data

## Figures and Tables

**Table 1:**
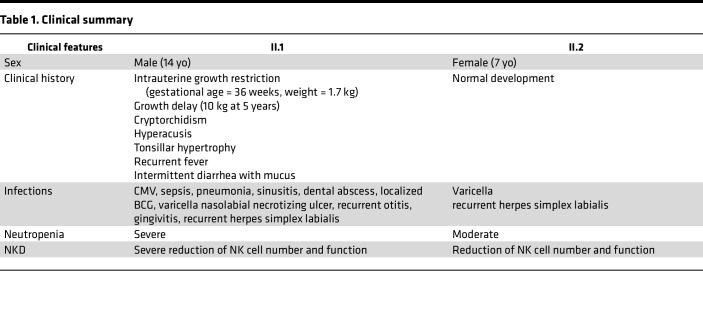
Clinical summary

**Table 2:**
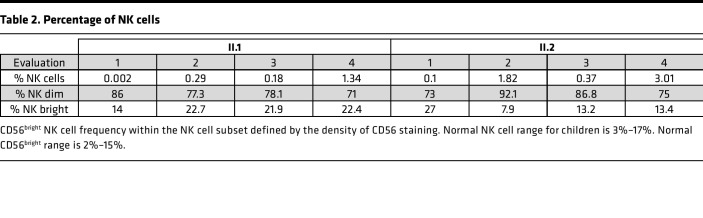
Percentage of NK cells

## Version 1 11/08/2022

Electronic publication
