# Supplementary material for: Partial loss-of-function mutations in GINS4 lead to NK cell deficiency with neutropenia
Source: JCI Insight. 2022 Nov 8;7(21):e154948. doi: 10.1172/jci.insight.154948 (PMC9675456; doi:10.1172/jci.insight.154948)

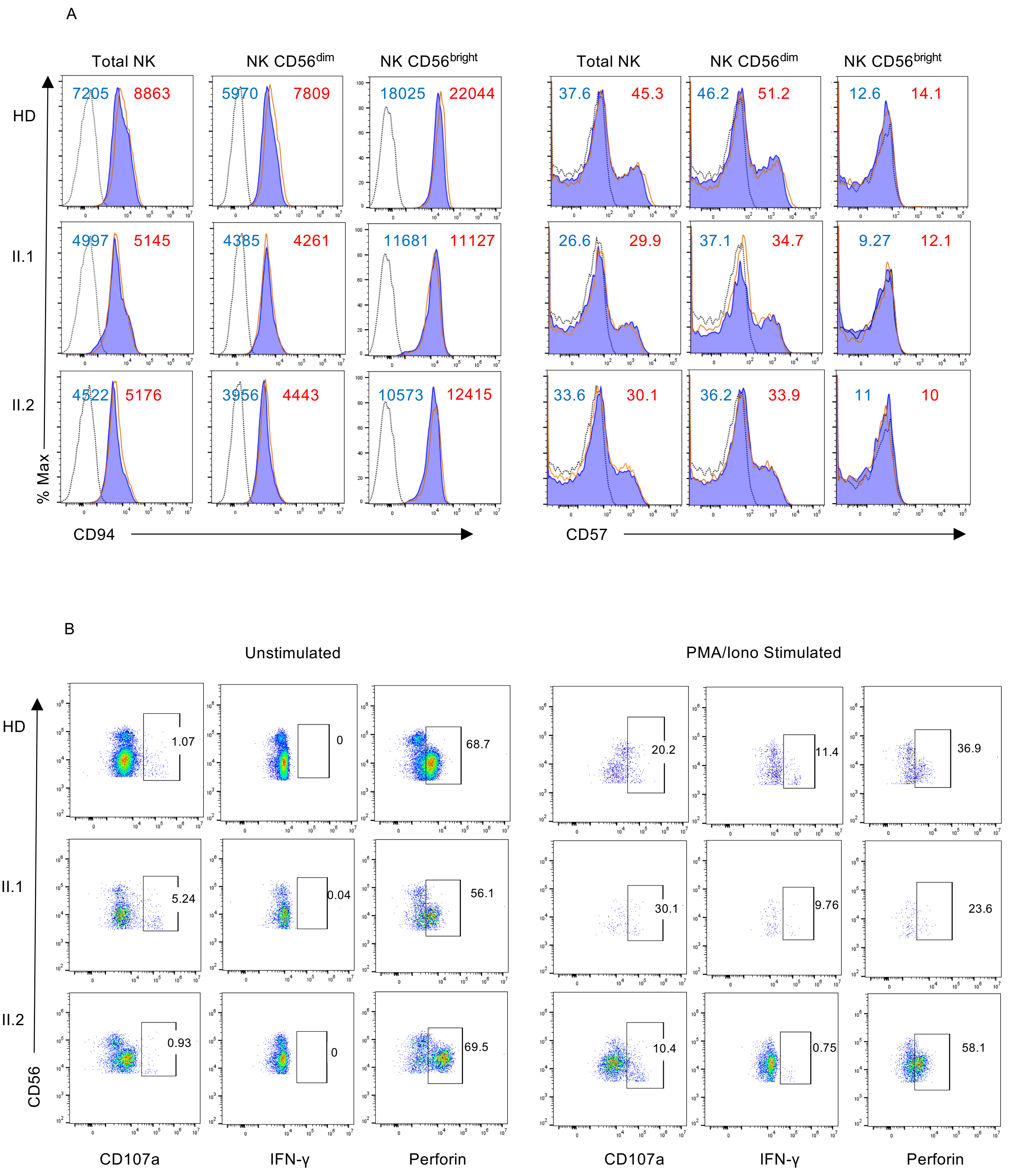

Supplemental Figure 1. NK cells phenotype and function. A) Expression of CD94 and CD57 on total NK cells, CD56^{dim}, and CD56^{bright} NK cells at baseline and after stimulation with 50ng/ml IL-15 for 48 hours showing FMO control (dotted line), resting PBMC (blue line), activated PBMC (red line). Mean fluorescence intensity (MFI) is indicated. B) Expression of CD107a, perforin and IFN-γ of resting NK cells and after 4 hours of PMA/Ionomycin stimulation. Healthy donor (HD, shipping control), II.1 and II.2 individuals are indicated.

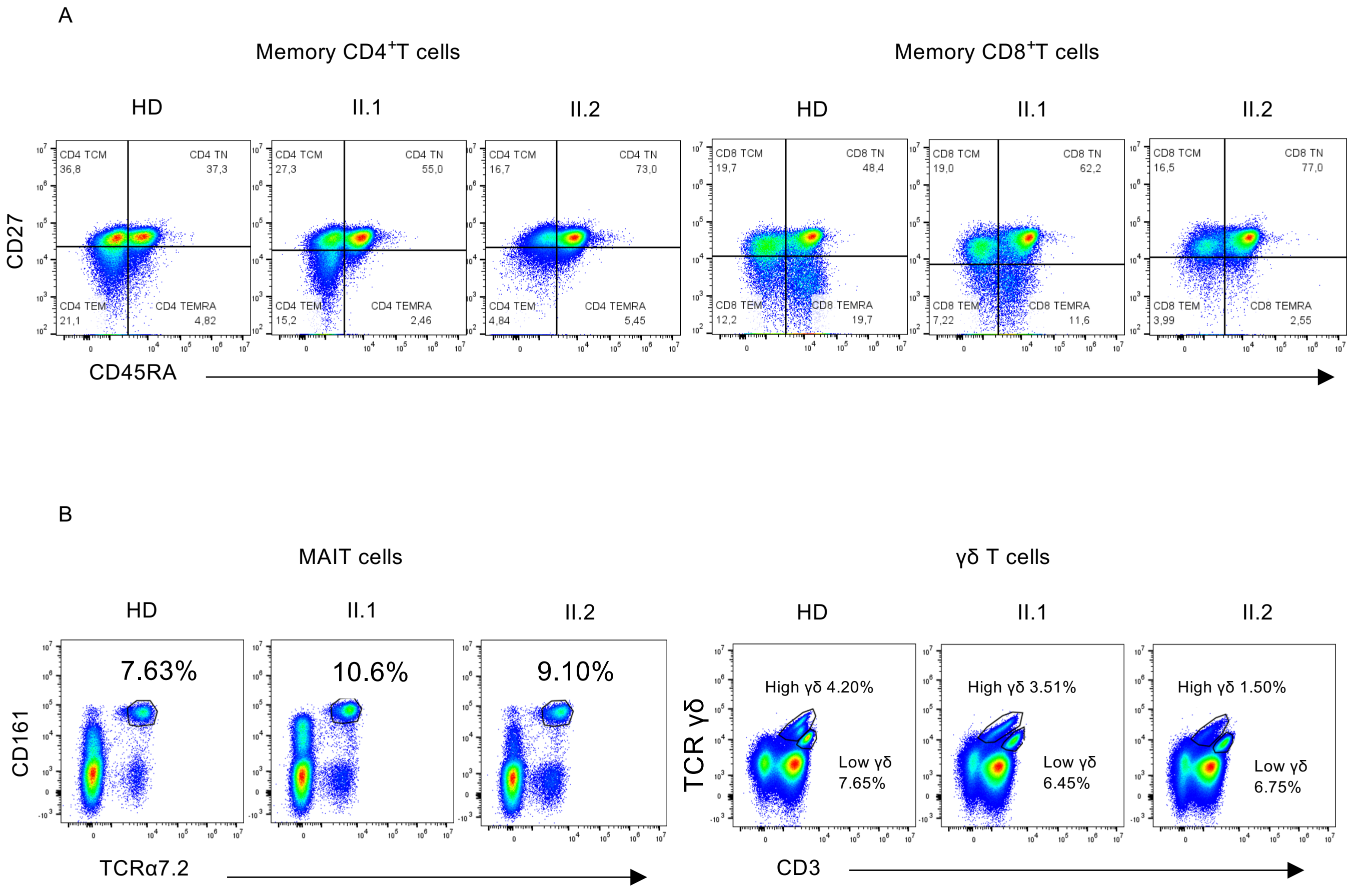

Supplemental Figure 2. Conventional T cells, MAIT cells and γδ T cells. A) Percentages of naïve T cells (TN), effector memory T cells (TEM), central memory T cells (TCM) and effector memory CD45RA$^+$T cells (TEMRA) of PBMC. B) Percentages of mucosal-associated invariant T (MAIT) cells and γδ T cells in healthy donor (HD, shipping control), II.1, II.2 individuals PBMC. MAIT cells were defined as CD3+CD8+CD161+TCRα7.2+ and γδ T cells as CD3+TCRγδ+.

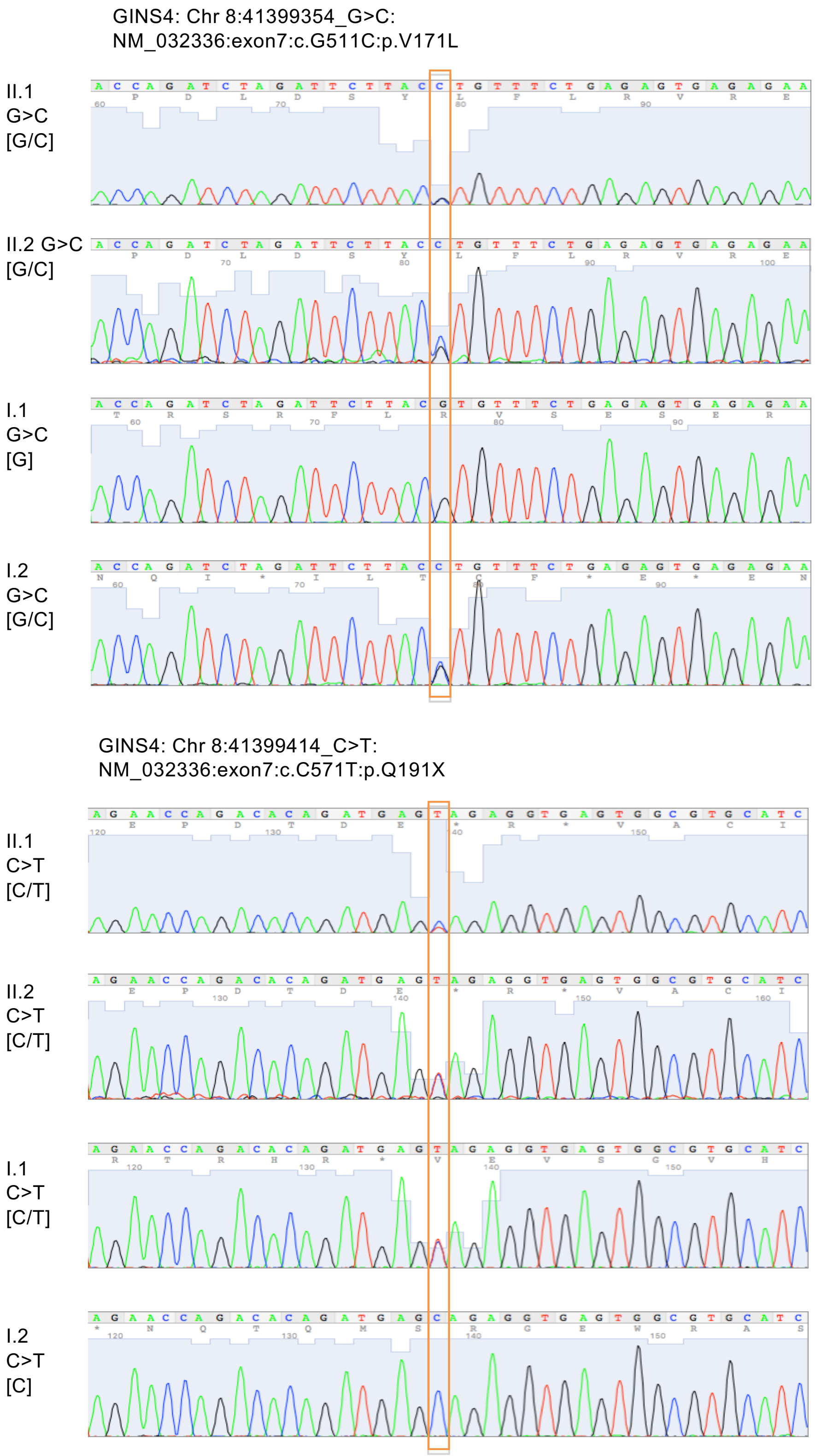

Supplemental Figure 3. Sanger sequencing confirmation in patient blood-derived DNA

A

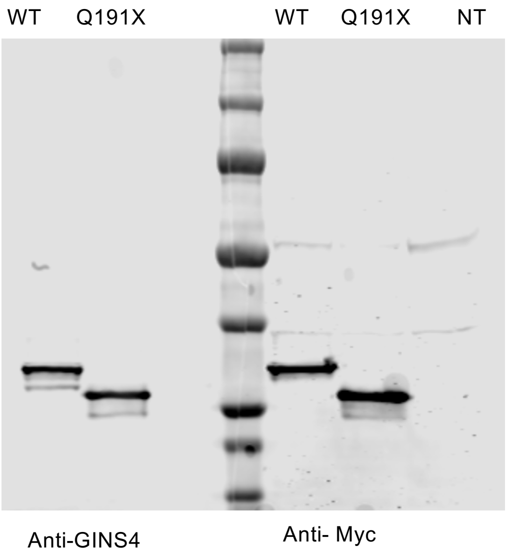

B

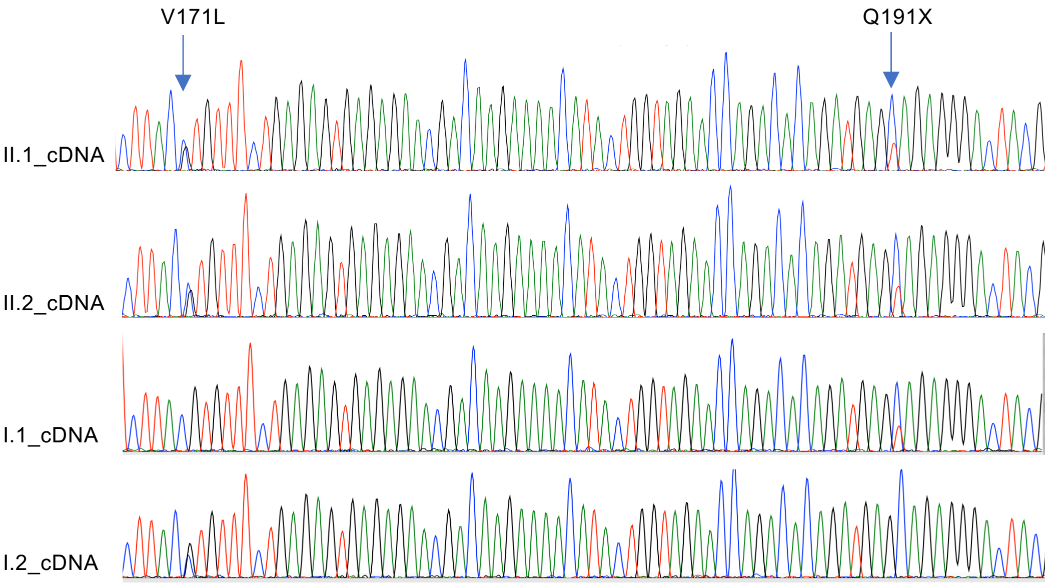

Supplemental Figure 4. A) Overexpression of the WT and stop codon variant Q191X in HEK293T. Immunoblotting with anti-GINS4 antibody and anti-Myc antibody, shows the antibody specificity to the full length protein and the stop codon allele-encoded variant. B) Stability of the stop codon at the mRNA level. The residue affecting the Q191X variant was identified in cDNA from the proband's cells and thus is present at the mRNA level, suggesting that if NMD occurs it is likely only partial.

A

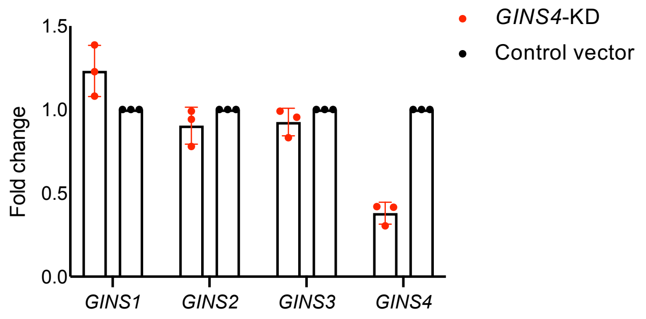

B

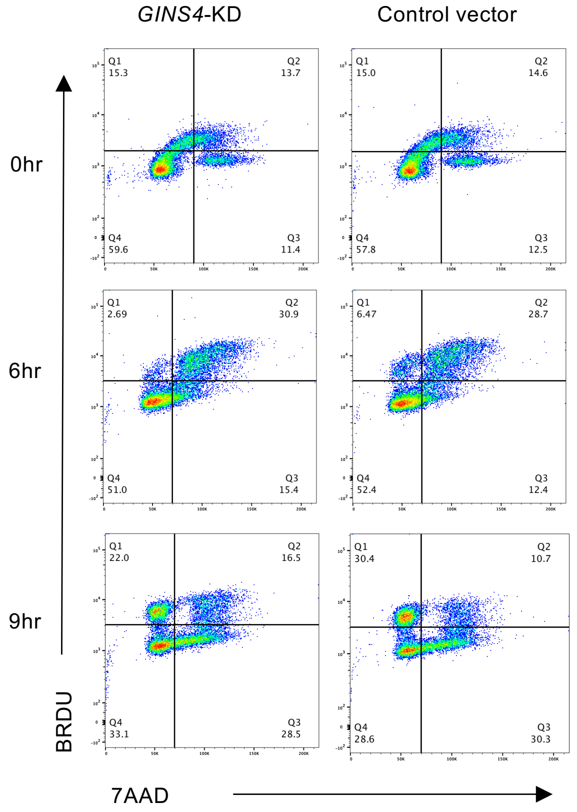

Supplemental Figure 5. A) Confirmatory qPCR analysis of *GINS4* expression in *GINS4* KD cell line. No off-target effect on other GINS are identified. B**)** Cell cycle progression analysis in *GINS4*-KD cell line. Representative flow cytometry plots of *GINS4*-KD and control vector cell lines shortly pulsed with BrdU and analyzed after 6 and 9 hours.

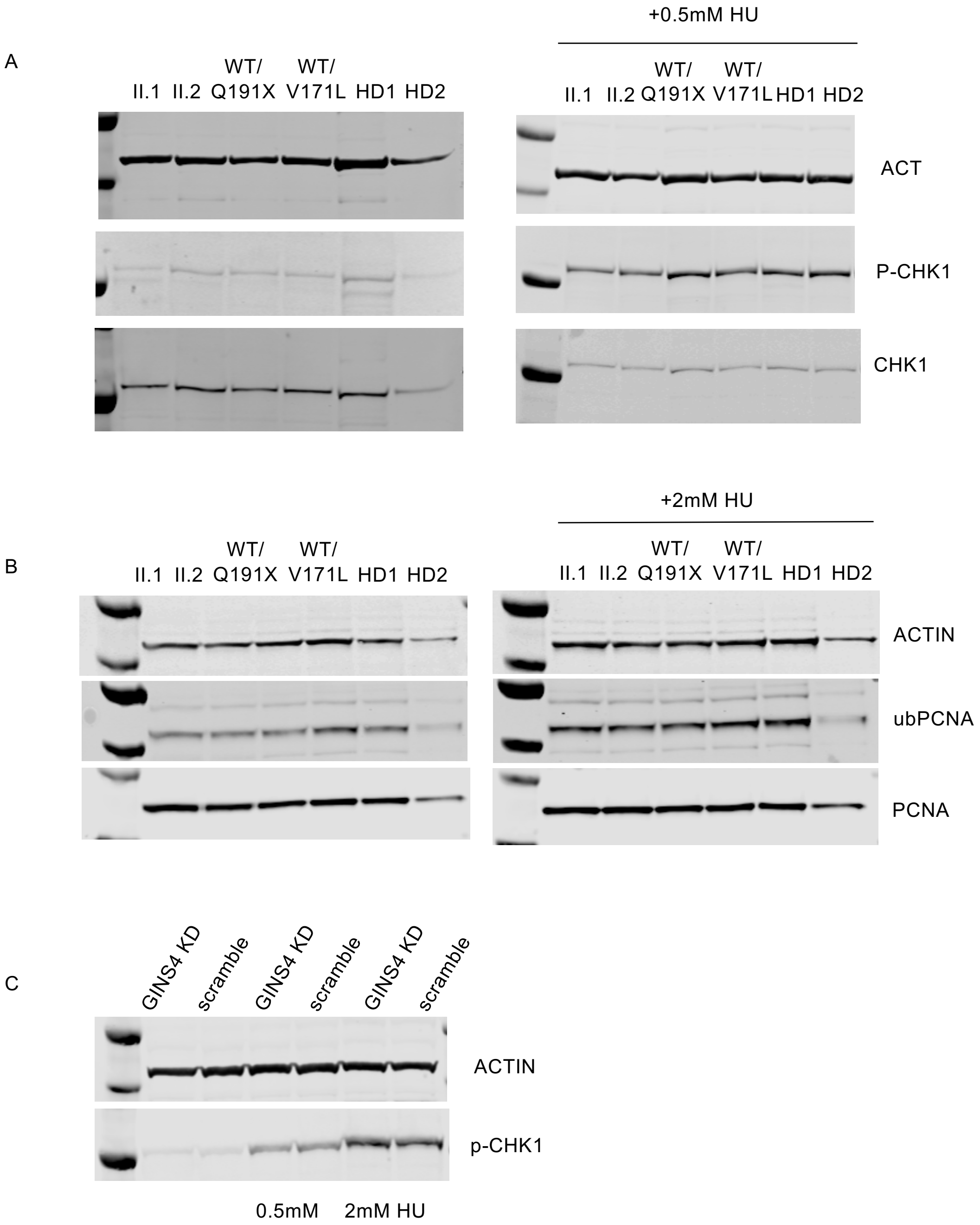

Supplemental Figure 6. individuals-derived cells show no impairments in DNA damage response. A) Phosphorylation of checkpoint 1 (p-CHK1) was evaluated at baseline and after 24hr of 0.5 hydroxyurea (HU) treatment in GINS4 family members and 2 HD. B) Ubiquitination of PCNA (ub-PCNA) was assessed at baseline and after 24hr of 2 μM hydroxyurea (HU) treatment in GINS4 family members and 2 HD. C) p-CHK1 was assessed at baseline and after 24hr of 0.5 and 2uM of HU treatment. Actin was used as a loading control. Data are representative of 2 independent experiments.

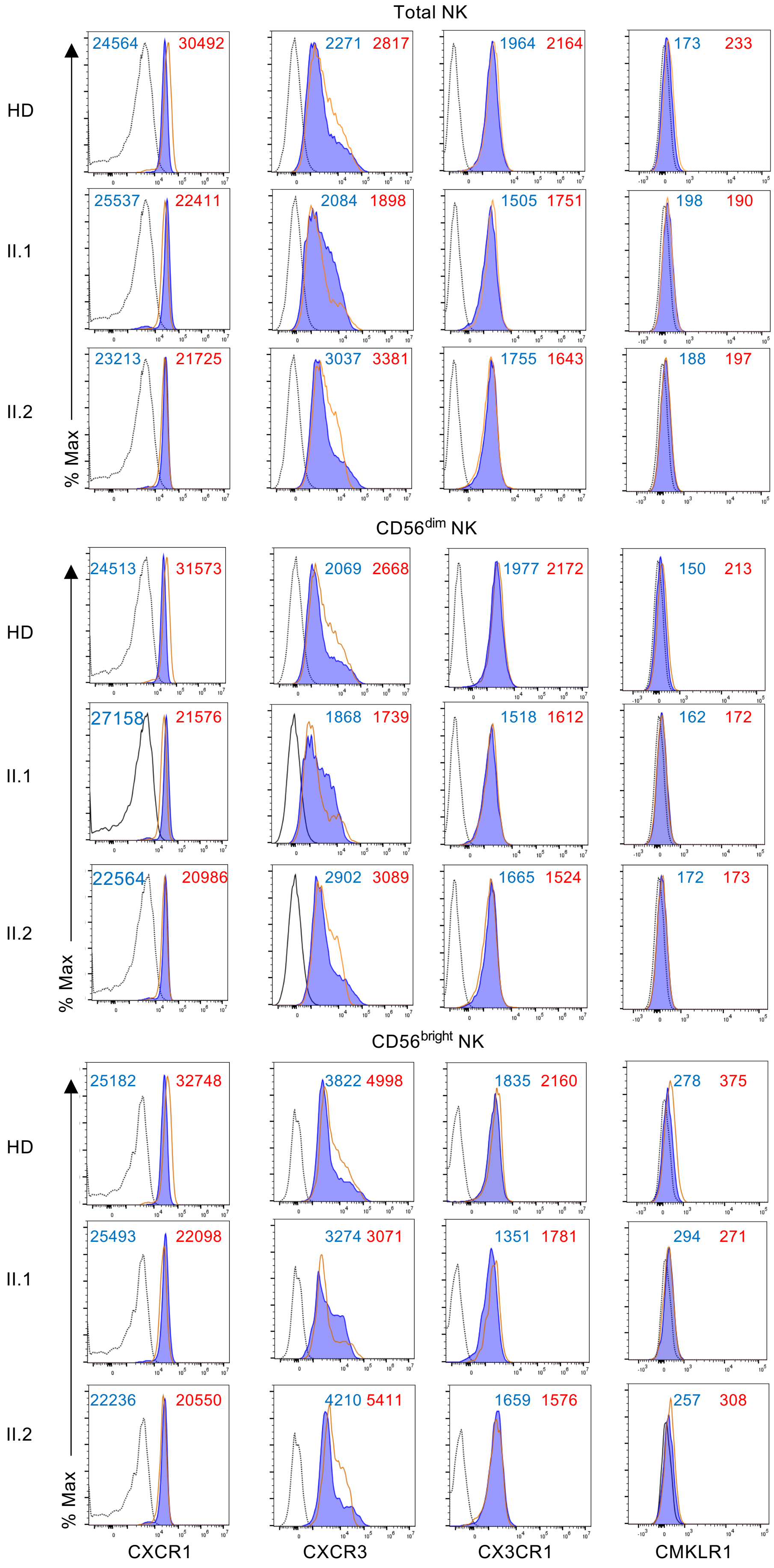

Supplemental Figure 7. Chemokine receptors expression in NK cell subsets. Expression of CXCR1, CXCR3, CX3CR1, AND CMKLR1 in total NK cells, CD56^{dim} NK, and CD56^{bright} NK at baseline and after stimulation with IL-15 for 48 hours. Dotted line: FMO control. Blue line: resting cells. Red line: PBMC stimulated with 50ng/ml IL-15. Mean fluorescence intensity (MFI) is indicated. Healthy donor (HD, shipping control), II.1, II.2 individuals are indicated.

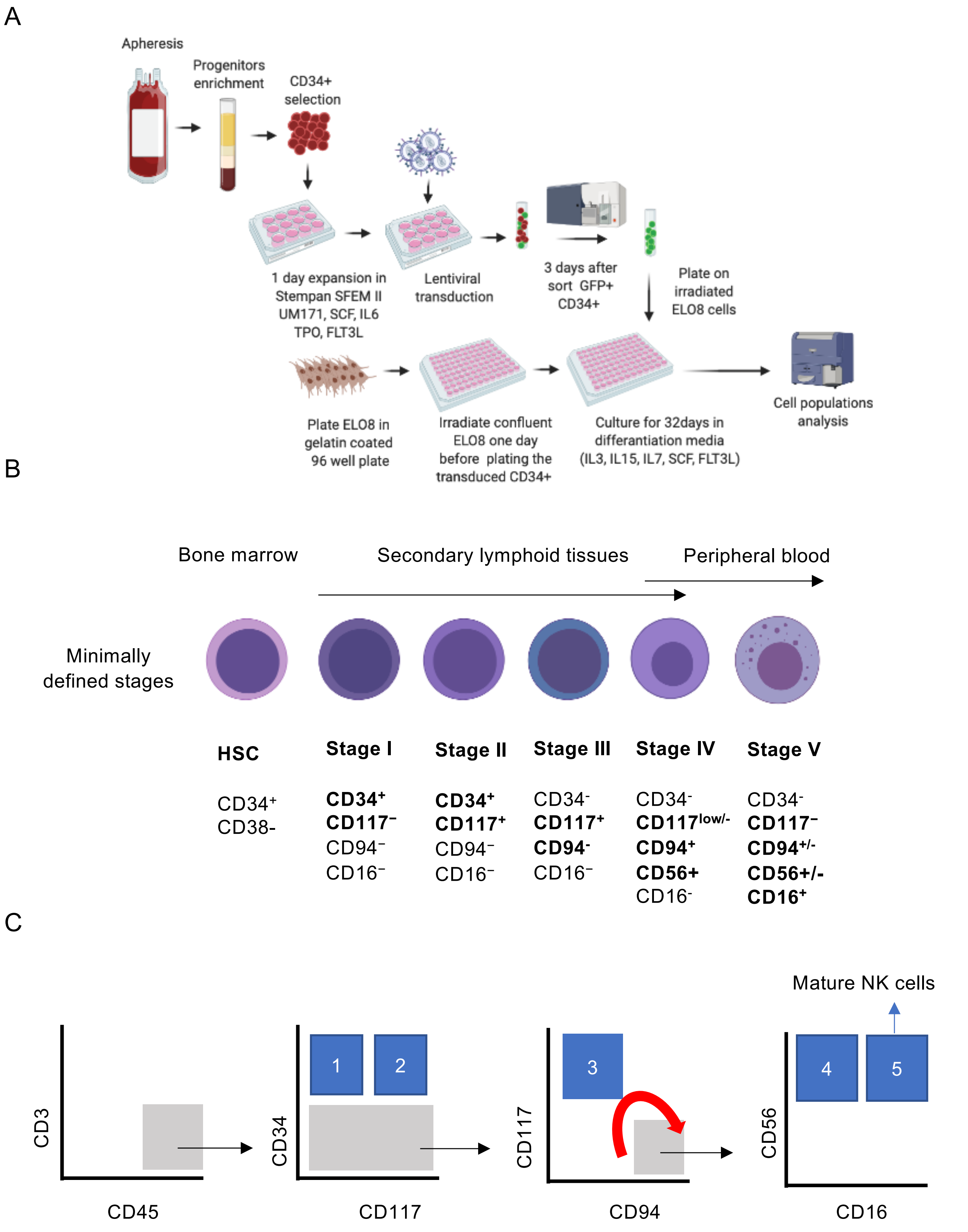

Supplemental Figure 8. A) CD34⁺ HSC precursors were isolated from apheresis products and expanded for one day in serum-free Stem Span II media supplemented with 35 nM UM171 and 100ng/ml of IL6, TPO, Flt3L, and SCF; then transduced with *GINS4* pGFP-C-shLenti or control vector pGFP-C-shLenti. Three days post transduction, GFP-positive cells were sorted and then co-cultured with irradiated EL08.1D2 stromal cells in the presence of cytokines as described in 'Methods'. Cells were harvested at day 32 and NK cell maturation was analyzed by FACS**.** B) Minimally defined stages of NK differentiation in vitro according to (Chichocki et al. 2010). C) Flow cytometry gating strategy that was applied to the differentiated cells on day 32. Schematic representation were created with biorender.com

Blood cell populations	II.1 (proband)	II.2 (sister)	I.1 (mother)
WBC	3690	4230	8280
**Neutrophils**	**270**	1050	4930
Lymphocytes	2730	2480	2490
Monocytes	650	640	610
Eosynophils	10	40	210
Platelets	432000	374000	354000
Total lymphocyte	69.51 % (2565)	60.91 % (2576)	30.19 % (2500)
$CD3^+$ T cells	54.57 % (2014)	41.33 % (1748)	16.88 % (1398)
$CD4^+$ T cells	28 % (763)	21.9 % (926)	11.64 % (964)
$CD8^+$ T cells	20.67 % (763)	13.73 % (581)	4.24 % (351)
$CD4^+$ /$CD8^+$ T cells	0.41 % (15)	0.06 % (3)	0.27 % (22)
$CD4^-$ /$CD8^-$ T cells	5.49 % (203)	5.64 % (239)	0.73 % (60)
CD4/CD8 ratio	1.35	1.6	2.75
$CD19^+$ B cells	13.81 % (510)	16.54 % (700)	7.38 % (611)
**$CD3^-$/$CD56^+$ NK cells**	1.13% (**42**)	3.04 % (**129**)	5.93 % (491)
Immunoglobulin levels			
IgG	7 yo -**1361** (453-916)<br>14 yo - 1471 (700-1600)	7 yo - 1283 (700-1600)	
IgA	7 yo - **346** (20-100)<br>14 yo - **453** (70-400)	7 yo - 248 (70-400)	
IgM	7 yo - 91 (19-146)<br>14 yo - 52 (40-230)	7 yo - 67 (40-230)	
IgE	14 yo - **82** (0-60)		

Supplemental table 1. Count and percentage of blood cells and immunoglobulons levels. Normal count range of NK cells=100-1400.

Supplemental Table 2. List of prioritized variants

GENE	chromosomal coordinates (hg19)	Zygosity	Mutation type	ExAC_ALL	Potential_InTrans	pLI	CADD score	coding change
LYST	1:235972859_BH9327-1_G>A	Het	nonsynonymous_SNV	.	FALSE	0,999978	26,4	LYST:NM_000081:exon5:c.C1259T:p.A420V
MDC1	6:30673730_BH9327-1_G>A	Het	nonsynonymous_SNV	.	FALSE	0,000942	10,2	MDC1:NM_014641:exon10:c.C3230T:p.T1077I
TCIRG1	11:67811770_BH9327-1_C>T	Het	stopgain_SNV	4,85E-05	FALSE	6,04E-06	24,9	TCIRG1:NM_006019:exon9:c.C979T:p.R327X
GINS4	8:41399354_BH9327-1_G>C	Het	nonsynonymous_SNV	.	TRUE	0,050473	32	GINS4:NM_032336:exon7:c.G511C:p.V171L
GINS4	8:41399414_BH9327-1_C>T	Het	stopgain_SNV	.	TRUE	0,050473	39	GINS4:NM_032336:exon7:c.C571T:p.Q191X
GSTP1	11:67352153_BH9327-1_C>T	Het	splicing	.	FALSE	0,115024	.	GSTP1(NM_000852:exon4:c.145-3C>T)
SLC2A1	1:43408997_BH9327-1_G>A	Het	splicing	0,0001	FALSE	0,939808	.	SLC2A1(NM_006516:exon3:c.19-5C>T)
EIF3B	7:2415100_BH9327-1_G>A	Het	nonsynonymous_SNV	.	FALSE	0,999971	25,8	EIF3B:NM_003751:exon14:c.G1966A:p.D656N
FGFR2	10:123239375_BH9327-1_G>A	Het	nonsynonymous_SNV	.	FALSE	0,999752	26,9	FGFR2:NM_023029:exon16:c.C2195T:p.T732I
MAP3K4	6:161510358_BH9327-1_A>G	Het	nonsynonymous_SNV	.	FALSE	0,999992	25,1	MAP3K4:NM_006724:exon11:c.A2828G:p.D943G
MAP3K6	1:27690566_BH9327-1_G>A	Het	nonsynonymous_SNV	2,55E-05	FALSE	4,91E-15	34	MAP3K6:NM_004672:exon5:c.C706T:p.R236W
MDM4	1:204518385_BH9327-1_C>T	Het	nonsynonymous_SNV	.	FALSE	0,99857	14,55	MDM4:NM_001278519:exon5:c.C379T:p.P127S
RNF19A	8:101272193_BH9327-1_C>T	Het	nonsynonymous_SNV	1,65E-05	FALSE	0,027076	24,2	RNF19A:NM_015435:exon10:c.G1715A:p.R572Q
SHC3	9:91661883_BH9327-1_G>C	Het	nonsynonymous_SNV	.	FALSE	0,457669	5,189	SHC3:NM_016848:exon8:c.C989G:p.T330R
STIM2	4:27004559_BH9327-1_G>A	Het	nonsynonymous_SNV	1,70E-05	FALSE	NA	28,5	STIM2:NM_001169117:exon7:c.G814A:p.A272T
TCF19	6:31129779_BH9327-1_C>G	Het	nonsynonymous_SNV	9,15E-06	FALSE	0,000233	0,312	TCF19:NM_001077511:exon3:c.C794G:p.T265S
SFPQ	1:35653649_BH9327-1_AATC>A	Het	nonframeshift_deletion	.	FALSE	0,999527	.	NONE(dist-NONE),NONE(dist-NONE),SFPQ:NM_005066:exon7:c.1737_1739del:p.579_580del
ATAD3A	1:1452592_BH9327-1_T>C	Hom	nonsynonymous_SNV	0,0004	FALSE	0,040131	0,002	ATAD3A:NM_018188:exon3:c.T328C:p.W110R
LILRA3	19:54802732_BH9327-1_C>T	Hom	nonsynonymous_SNV	.	FALSE	0,002925	.	LILRA3:NM_006865:exon5:c.G709A:p.A237T
KMT2C	7:151860023_BH9327-1_A>G	Het	nonsynonymous_SNV	0,0021	FALSE	1	23,4	KMT2C:NM_170606:exon43:c.T10639C:p.S3547P
KMT2C	7:151935797_BH9327-1_TG>T	Het	frameshift_deletion	0,001	FALSE	1	.	NONE(dist-NONE),NONE(dist-NONE),KMT2C:NM_170606:exon15:c.2646delC:p.I882fs
MST1L	1:17086716_BH9327-1_G>A	Het	nonsynonymous_SNV	.	TRUE	NA	.	MST1L:NM_001271733:exon5:c.C527T:p.T176I
MST1L	1:17086974_BH9327-1_G>A	Het	nonsynonymous_SNV	.	TRUE	NA	.	MST1L:NM_001271733:exon4:c.C350T:p.T117M
SOX18	20:62679616_BH9327-1_C>T	Het	nonsynonymous_SNV	2,61E-05	TRUE	NA	23,9	SOX18:NM_018419:exon2:c.G1058A:p.G353D
SOX18	20:62680265_BH9327-1_C>T	Het	nonsynonymous_SNV	0,001	TRUE	NA	28,6	SOX18:NM_018419:exon2:c.G409A:p.E137K
TNXB	6:32021362_BH9327-1_A>G	Het	nonsynonymous_SNV	0,0061	TRUE	0,774154	0,003	TNXB:NM_019105:exon25:c.T8588C:p.M2863T
TNXB	6:32038185_BH9327-1_C>T	Het	nonsynonymous_SNV	5,17E-05	TRUE	0,774154	8,068	TNXB:NM_019105:exon14:c.G4997A:p.R1666Q
ZNF678	1:227842964_BH9327-1_G>A	Het	nonsynonymous_SNV	1,69E-05	FALSE	4,67E-07	0,041	ZNF678:NM_178549:exon4:c.G1178A:p.S393N
ZNF678	1:227842973_BH9327-1_G>A	Het	nonsynonymous_SNV	3,40E-05	FALSE	4,67E-07	9,631	ZNF678:NM_178549:exon4:c.G1187A:p.R396K

Supplemental Table 3. Customized immune panel

Probe Name	Accession #	Class Name
ABCB1	NM_000927.3	Endogenous
ABL1	NM_005157.3	Endogenous
ADA	NM_000022.2	Endogenous
AHR	NM_001621.3	Endogenous
AICDA	NM_020661.1	Endogenous
AIRE	NM_000383.2	Endogenous
APP	NM_000484.3	Endogenous
ARG1	NM_000045.2	Endogenous
ARG2	NM_001172.3	Endogenous
ARHGDIB	NM_001175.4	Endogenous
ATG10	NM_001131028.1	Endogenous
ATG12	NM_004707.2	Endogenous
ATG16L1	NM_198890.2	Endogenous
ATG5	NM_004849.2	Endogenous
ATG7	NM_001136031.2	Endogenous
ATM	NM_000051.3	Endogenous
ATR	NM_001184.2	Endogenous
B2M	NM_004048.2	Endogenous
B3GAT1	NM_018644.3	Endogenous
BAD	NM_004322.3	Endogenous
BATF	NM_006399.3	Endogenous
BATF3	NM_018664.2	Endogenous
BAX	NM_138761.3	Endogenous
BCAP31	NM_005745.7	Endogenous
BCL10	NM_003921.2	Endogenous
BCL2	NM_000657.2	Endogenous
BCL2L11	NM_138621.4	Endogenous
BCL3	NM_005178.2	Endogenous
BCL6	NM_001706.2	Endogenous
CD244	NM_016382.2	Endogenous
BLNK	NM_013314.2	Endogenous
BRCA1	NM_007305.2	Endogenous
BST1	NM_004334.2	Endogenous
BST2	NM_004335.2	Endogenous
BTK	NM_000061.1	Endogenous
BTLA	NM_181780.2	Endogenous
C14orf166	NM_016039.2	Endogenous
C1QA	NM_015991.2	Endogenous
C1QB	NM_000491.3	Endogenous
C1QBP	NM_001212.3	Endogenous

C1R	NM_001733.4	Endogenous
C1S	NM_001734.2	Endogenous
C2	NM_000063.3	Endogenous
C3	NM_000064.2	Endogenous
C4A/B	NM_007293.2	Endogenous
C4BPA	NM_000715.3	Endogenous
C5	NM_001735.2	Endogenous
C6	NM_000065.2	Endogenous
C7	NM_000587.2	Endogenous
C8A	NM_000562.2	Endogenous
C8B	NM_000066.2	Endogenous
C8G	NM_000606.2	Endogenous
C9	NM_001737.3	Endogenous
CAMP	NM_004345.3	Endogenous
CARD9	NM_052813.4	Endogenous
CASP1	NM_001223.3	Endogenous
CASP10	NM_032977.3	Endogenous
CASP2	NM_032982.2	Endogenous
FAS	NM_000043.3	Endogenous
CASP8	NM_001228.4	Endogenous
CCBP2	NM_001296.3	Endogenous
FCER1A	NM_002001.2	Endogenous
CCL13	NM_005408.2	Endogenous
FCGR1A/B	NM_000566.3	Endogenous
CCL16	NM_004590.2	Endogenous
FCGR2A/C	NM_201563.4	Endogenous
CCL19	NM_006274.2	Endogenous
FCGR3A/B	NM_000570.4	Endogenous
CCL20	NM_004591.1	Endogenous
CCL22	NM_002990.3	Endogenous
CCL23	NM_145898.1	Endogenous
CCL24	NM_002991.2	Endogenous
CCL26	NM_006072.4	Endogenous
IL12RB1	NM_005535.1	Endogenous
CCL4	NM_002984.2	Endogenous
CCL5	NM_002985.2	Endogenous
CCL7	NM_006273.2	Endogenous
CCL8	NM_005623.2	Endogenous
CCND3	NM_001760.2	Endogenous
CCR1	NM_001295.2	Endogenous
CCR10	NM_016602.2	Endogenous
CCR2	NM_001123041.2	Endogenous

CCR5	NM_000579.1	Endogenous
CCR6	NM_031409.2	Endogenous
CCR7	NM_001838.2	Endogenous
CCR8	NM_005201.2	Endogenous
CCRL1	NM_016557.2	Endogenous
CCRL2	NM_003965.4	Endogenous
CD14	NM_000591.2	Endogenous
CD160	NM_007053.2	Endogenous
CD163	NM_004244.4	Endogenous
CD164	NM_006016.4	Endogenous
CD19	NM_001770.4	Endogenous
CD1A	NM_001763.2	Endogenous
CD1D	NM_001766.3	Endogenous
CD2	NM_001767.3	Endogenous
CD209	NM_021155.2	Endogenous
CD22	NM_001771.2	Endogenous
CD24	NM_013230.2	Endogenous
IL18RAP	NM_003853.2	Endogenous
CD247	NM_198053.1	Endogenous
CD27	NM_001242.4	Endogenous
CD274	NM_014143.3	Endogenous
CD276	NM_001024736.1	Endogenous
CD28	NM_001243078.1	Endogenous
CD34	NM_001025109.1	Endogenous
CD36	NM_001001548.2	Endogenous
CD3D	NM_000732.4	Endogenous
CD3E	NM_000733.2	Endogenous
CD3EAP	NM_012099.1	Endogenous
CD4	NM_000616.4	Endogenous
CD40	NM_001250.4	Endogenous
CD40LG	NM_000074.2	Endogenous
CD44	NM_001001392.1	Endogenous
CD45R0	NM_080921.3	Endogenous
CD45RA	NM_002838.4	Endogenous
CD45RB	ENST00000367367.1	Endogenous
CD46	NM_172350.1	Endogenous
IL21R	NM_021798.2	Endogenous
CD5	NM_014207.2	Endogenous
CD53	NM_001040033.1	Endogenous
CD55	NM_000574.3	Endogenous
CD58	NM_001779.2	Endogenous
CD59	NM_000611.4	Endogenous

CD6	NM_006725.3	Endogenous
CD7	NM_006137.6	Endogenous
CD70	NM_001252.2	Endogenous
CD74	NM_001025159.1	Endogenous
CD79A	NM_001783.3	Endogenous
CD79B	NM_021602.2	Endogenous
CD80	NM_005191.3	Endogenous
CD81	NM_004356.3	Endogenous
CD82	NM_002231.3	Endogenous
CD83	NM_004233.3	Endogenous
CD86	NM_175862.3	Endogenous
CD8A	NM_001768.5	Endogenous
CD8B	NM_004931.3	Endogenous
CD9	NM_001769.2	Endogenous
CD96	NM_005816.4	Endogenous
CD97	NM_078481.2	Endogenous
CD99	NM_002414.3	Endogenous
CDH5	NM_001795.3	Endogenous
CDK2	NM_001798.2	Endogenous
CDKN1A	NM_000389.2	Endogenous
CEACAM1	NM_001712.3	Endogenous
CEACAM6	NM_002483.4	Endogenous
CEACAM8	NM_001816.3	Endogenous
CEBPB	NM_005194.2	Endogenous
CFB	NM_001710.5	Endogenous
CFD	NM_001928.2	Endogenous
CFH	NM_001014975.2	Endogenous
CFI	NM_000204.3	Endogenous
CFP	NM_002621.2	Endogenous
IL2RB	NM_000878.2	Endogenous
ITGB2	NM_000211.2	Endogenous
CISH	NM_145071.2	Endogenous
CLEC4A	NM_194448.2	Endogenous
CLEC4E	NM_014358.2	Endogenous
CLEC5A	NM_013252.2	Endogenous
CLEC6A	NM_001007033.1	Endogenous
CLEC7A	NM_197954.2	Endogenous
CLU	NM_001831.2	Endogenous
KLRB1	NM_002258.2	Endogenous
CR1	NM_000651.4	Endogenous
CR2	NM_001006658.1	Endogenous
CRADD	NM_003805.3	Endogenous

CSF1	NM_000757.4	Endogenous
CSF1R	NM_005211.2	Endogenous
KLRD1	NM_002262.3	Endogenous
CSF2RB	NM_000395.2	Endogenous
CSF3R	NM_156038.2	Endogenous
CTLA4-TM	NM_005214.3	Endogenous
CTLA4_all	NM_005214.3	Endogenous
CTNNB1	NM_001098210.1	Endogenous
CTSC	NM_001814.4	Endogenous
CTSG	NM_001911.2	Endogenous
CTSS	NM_004079.3	Endogenous
CUL9	NM_015089.2	Endogenous
CX3CL1	NM_002996.3	Endogenous
KLRG1	NM_005810.3	Endogenous
CXCL1	NM_001511.1	Endogenous
CXCL10	NM_001565.1	Endogenous
CXCL11	NM_005409.4	Endogenous
CXCL12	NM_000609.5	Endogenous
CXCL13	NM_006419.2	Endogenous
KLRG2	NM_198508.2	Endogenous
CXCL9	NM_002416.1	Endogenous
KLRK1	NM_007360.1	Endogenous
CXCR2	NM_001557.2	Endogenous
TNFRSF10C	NM_003841.3	Endogenous
CXCR4	NM_003467.2	Endogenous
CXCR6	NM_006564.1	Endogenous
CYBB	NM_000397.3	Endogenous
Cyclin D	NM_001142326.1	Endogenous
Cyclin E	NM_001238.1	Endogenous
DEFB1	NM_005218.3	Endogenous
DEFB103A	NM_001081551.2	Endogenous
DEFB103B	NM_018661.3	Endogenous
DEFB4A	NM_004942.2	Endogenous
DPP4	NM_001935.3	Endogenous
DUSP4	NM_057158.2	Endogenous
E2F1	NM_005225.1	Endogenous
EBI3	NM_005755.2	Endogenous
EDNRB	NM_003991.2	Endogenous
EGR1	NM_001964.2	Endogenous
EGR2	NM_000399.3	Endogenous
ENTPD1	NM_001098175.1	Endogenous
EOMES	NM_005442.2	Endogenous

ETS1	NM_005238.3	Endogenous
FADD	NM_003824.2	Endogenous
BID	NM_001196.2	Endogenous
FCAR	NM_133280.1	Endogenous
CASP3	NM_032991.2	Endogenous
FCER1G	NM_004106.1	Endogenous
CCL11	NM_002986.2	Endogenous
FCGR2A	NM_021642.3	Endogenous
CCL15	NM_032965.3	Endogenous
FCGR2B	NM_001002273.1	Endogenous
CCL18	NM_002988.2	Endogenous
FCGRT	NM_004107.4	Endogenous
FKBP5	NM_001145775.1	Endogenous
FN1	NM_212482.1	Endogenous
FOXP3	NM_014009.3	Endogenous
FYN	NM_002037.3	Endogenous
GADD45A	NM_001924.2	Endogenous
GATA3	NM_001002295.1	Endogenous
GBP1	NM_002053.1	Endogenous
GBP5	NM_052942.3	Endogenous
GFI1	NM_005263.2	Endogenous
GNLY	NM_006433.2	Endogenous
GP1BB	NM_000407.4	Endogenous
GPI	NM_000175.2	Endogenous
GPR183	NM_004951.3	Endogenous
GZMA	NM_006144.2	Endogenous
GZMB	NM_004131.3	Endogenous
GZMK	NM_002104.2	Endogenous
HAMP	NM_021175.2	Endogenous
HAVCR2	NM_032782.3	Endogenous
HFE	NM_139011.2	Endogenous
HLA-A	NM_002116.5	Endogenous
HLA-B	NM_005514.6	Endogenous
HLA-C	NM_002117.4	Endogenous
HLA-DMA	NM_006120.3	Endogenous
HLA-DMB	NM_002118.3	Endogenous
HLA-DOB	NM_002120.3	Endogenous
HLA-DPA1	NM_033554.2	Endogenous
HLA-DPB1	NM_002121.4	Endogenous
HLA-DQA1	NM_002122.3	Endogenous
HLA-DQB1	NM_002123.3	Endogenous
HLA-DRA	NM_019111.3	Endogenous

HLA-DRB1	NM_002124.2	Endogenous
HLA-DRB3	NM_022555.3	Endogenous
HRAS	NM_005343.2	Endogenous
CCL2	NM_002982.3	Endogenous
ICAM2	NM_000873.3	Endogenous
ICAM3	NM_002162.3	Endogenous
ICAM4	NM_001039132.1	Endogenous
ICAM5	NM_003259.3	Endogenous
ICOS	NM_012092.2	Endogenous
ICOSLG	NM_015259.4	Endogenous
IDO1	NM_002164.3	Endogenous
CCL3	NM_002983.2	Endogenous
IFI35	NM_005533.3	Endogenous
IFIH1	NM_022168.2	Endogenous
IFIT2	NM_001547.4	Endogenous
IFITM1	NM_003641.3	Endogenous
IFNA1/13	NM_024013.1	Endogenous
CD48	NM_001778.2	Endogenous
IFNAR1	NM_000629.2	Endogenous
IFNAR2	NM_000874.3	Endogenous
CHUK	NM_001278.3	Endogenous
IFNG	NM_000619.2	Endogenous
IFNGR1	NM_000416.1	Endogenous
IGF2R	NM_000876.1	Endogenous
IKBKAP	NM_003640.3	Endogenous
IKBKB	NM_001556.1	Endogenous
IKBKE	NM_014002.2	Endogenous
IKBKG	NM_003639.2	Endogenous
IKZF1	NM_006060.3	Endogenous
IKZF2	NM_016260.2	Endogenous
CIITA	NM_000246.3	Endogenous
IL10	NM_000572.2	Endogenous
IL10RA	NM_001558.2	Endogenous
IL11RA	NM_147162.1	Endogenous
IL12A	NM_000882.2	Endogenous
IL12B	NM_002187.2	Endogenous
CMKLR1	NM_004072.1	Endogenous
IL13	NM_002188.2	Endogenous
IL13RA1	NM_001560.2	Endogenous
IL15	NM_172174.1	Endogenous
IL16	NM_004513.4	Endogenous
IL17A	NM_002190.2	Endogenous

IL17B	NM_014443.2	Endogenous
IL17F	NM_052872.3	Endogenous
IL18	NM_001562.2	Endogenous
IL18R1	NM_003855.2	Endogenous
CSF2	NM_000758.2	Endogenous
IL19	NM_013371.3	Endogenous
IL1A	NM_000575.3	Endogenous
IL1B	NM_000576.2	Endogenous
IL1R1	NM_000877.2	Endogenous
IL1R2	NM_173343.1	Endogenous
IL1RAP	NM_002182.2	Endogenous
IL1RL1	NM_016232.4	Endogenous
IL1RL2	NM_003854.2	Endogenous
IL1RN	NM_000577.3	Endogenous
IL2	NM_000586.2	Endogenous
IL20	NM_018724.3	Endogenous
IL21	NM_021803.2	Endogenous
CX3CR1	NM_001337.3	Endogenous
IL22	NM_020525.4	Endogenous
IL22RA2	NM_181310.1	Endogenous
IL23A	NM_016584.2	Endogenous
IL23R	NM_144701.2	Endogenous
IL26	NM_018402.1	Endogenous
IL27	NM_145659.3	Endogenous
IL28A	NM_172138.1	Endogenous
IL28A/B	NM_172139.2	Endogenous
IL29	NM_172140.1	Endogenous
IL2RA	NM_000417.1	Endogenous
CXCL2	NM_002089.3	Endogenous
IL2RG	NM_000206.1	Endogenous
IL3	NM_000588.3	Endogenous
IL32	NM_001012633.1	Endogenous
IL4	NM_000589.2	Endogenous
IL4R	NM_000418.2	Endogenous
IL5	NM_000879.2	Endogenous
IL6	NM_000600.1	Endogenous
IL6R	NM_000565.2	Endogenous
IL6ST	NM_002184.2	Endogenous
IL7	NM_000880.2	Endogenous
IL7R	NM_002185.2	Endogenous
IL8	NM_000584.2	Endogenous
IL9	NM_000590.1	Endogenous

ILF3	NM_001137673.1	Endogenous
IRAK1	NM_001569.3	Endogenous
IRAK2	NM_001570.3	Endogenous
IRAK3	NM_007199.1	Endogenous
IRAK4	NM_016123.1	Endogenous
IRF1	NM_002198.1	Endogenous
IRF3	NM_001571.5	Endogenous
IRF4	NM_002460.1	Endogenous
IRF5	NM_002200.3	Endogenous
IRF7	NM_001572.3	Endogenous
CXCR1	NM_000634.2	Endogenous
IRGM	NM_001145805.1	Endogenous
ITGA2B	NM_000419.3	Endogenous
ITGA4	NM_000885.4	Endogenous
ITGA5	NM_002205.2	Endogenous
ITGA6	NM_000210.1	Endogenous
ITGAE	NM_002208.4	Endogenous
ITGAL	NM_002209.2	Endogenous
ITGAM	NM_000632.3	Endogenous
ITGAX	NM_000887.3	Endogenous
ITGB1	NM_033666.2	Endogenous
CXCR3	NM_001504.1	Endogenous
ITLN1	NM_017625.2	Endogenous
ITLN2	NM_080878.2	Endogenous
JAK1	NM_002227.1	Endogenous
JAK2	NM_004972.2	Endogenous
JAK3	NM_000215.2	Endogenous
KCNJ2	NM_000891.2	Endogenous
KIR3DL1	NM_013289.2	Endogenous
KIR3DL2	NM_006737.2	Endogenous
KIR3DL3	NM_153443.3	Endogenous
KIR_Activating_Subgroup_1	NM_001083539.1	Endogenous
KIR_Activating_Subgroup_2	NM_014512.1	Endogenous
KIR_Inhibiting_Subgroup_1	NM_014218.2	Endogenous
KIR_Inhibiting_Subgroup_2	NM_014511.3	Endogenous
KIT	NM_000222.2	Endogenous
KLRAP1	NR_028045.1	Endogenous
ICAM1	NM_000201.2	Endogenous
KLRC1	NM_002259.3	Endogenous
KLRC2	NM_002260.3	Endogenous
KLRC3	NM_007333.2	Endogenous
KLRC4	NM_013431.2	Endogenous

IFI16	NM_005531.1	Endogenous
KLRF1	NM_016523.1	Endogenous
KLRF2	NM_001190765.1	Endogenous
IFNA2	NM_000605.3	Endogenous
IFNB1	NM_002176.2	Endogenous
IKZF3	NM_183232.2	Endogenous
LAG3	NM_002286.5	Endogenous
LAIR1	NM_002287.3	Endogenous
LAMP3	NM_014398.3	Endogenous
IRF8	NM_002163.2	Endogenous
LCP2	NM_005565.3	Endogenous
LEF1	NM_016269.3	Endogenous
LGALS3	NM_001177388.1	Endogenous
LIF	NM_002309.3	Endogenous
LILRA1	NM_006863.1	Endogenous
LILRA2	NM_006866.2	Endogenous
LILRA3	NM_006865.3	Endogenous
LILRA4	NM_012276.3	Endogenous
LILRA5	NM_181879.2	Endogenous
LILRA6	NM_024318.2	Endogenous
LILRB1	NM_001081637.1	Endogenous
LILRB2	NM_005874.1	Endogenous
LILRB3	NM_006864.2	Endogenous
LILRB4	NM_001081438.1	Endogenous
LILRB5	NM_001081442.1	Endogenous
LITAF	NM_004862.3	Endogenous
LTA	NM_000595.2	Endogenous
LTB4R	NM_181657.3	Endogenous
LTB4R2	NM_019839.4	Endogenous
LTBR	NM_002342.1	Endogenous
LTF	NM_002343.2	Endogenous
LY96	NM_015364.2	Endogenous
MAF	NM_005360.4	Endogenous
MALT1	NM_006785.2	Endogenous
MAP4K1	NM_007181.3	Endogenous
MAP4K2	NM_004579.2	Endogenous
MAP4K4	NM_004834.3	Endogenous
MAPK1	NM_138957.2	Endogenous
MAPK11	NM_002751.5	Endogenous
MAPK14	NM_001315.1	Endogenous
MAPKAPK2	NM_004759.3	Endogenous
MARCO	NM_006770.3	Endogenous

MASP1	NM_139125.3	Endogenous
MASP2	NM_139208.1	Endogenous
MBL2	NM_000242.2	Endogenous
MBP	NM_002385.2	Endogenous
MCL1	NM_021960.3	Endogenous
MDM2	NM_001145337.1	Endogenous
MIF	NM_002415.1	Endogenous
MME	NM_000902.2	Endogenous
MR1	NM_001531.2	Endogenous
MRC1	NM_002438.2	Endogenous
MS4A1	NM_152866.2	Endogenous
MSR1	NM_002445.3	Endogenous
MUC1	NM_001018017.1	Endogenous
MX1	NM_002462.2	Endogenous
MYD88	NM_002468.3	Endogenous
NCAM1	NM_000615.5	Endogenous
NCF4	NM_000631.4	Endogenous
NCR1	NM_004829.5	Endogenous
NFATC1	NM_172389.1	Endogenous
NFATC2	NM_012340.3	Endogenous
NFATC3	NM_004555.2	Endogenous
NFIL3	NM_005384.2	Endogenous
NFKB1	NM_003998.2	Endogenous
NFKB2	NM_002502.2	Endogenous
NFKBIA	NM_020529.1	Endogenous
NFKBIZ	NM_001005474.1	Endogenous
NLRP3	NM_001079821.2	Endogenous
NOD1	NM_006092.1	Endogenous
NOD2	NM_022162.1	Endogenous
NOS2	NM_000625.4	Endogenous
NOTCH1	NM_017617.3	Endogenous
NOTCH2	NM_024408.3	Endogenous
NT5E	NM_002526.2	Endogenous
PAX5	NM_016734.1	Endogenous
PDCD1	NM_005018.1	Endogenous
PDCD1LG2	NM_025239.3	Endogenous
PDCD2	NM_144781.2	Endogenous
PDGFB	NM_033016.2	Endogenous
PDGFRB	NM_002609.3	Endogenous
PECAM1	NM_000442.3	Endogenous
PIGR	NM_002644.2	Endogenous
PLA2G2A	NM_000300.2	Endogenous

PLA2G2E	NM_014589.1	Endogenous
PLAU	NM_002658.2	Endogenous
PLAUR	NM_001005376.1	Endogenous
PML	NM_002675.3	Endogenous
POU2F2	NM_002698.2	Endogenous
PPARG	NM_015869.3	Endogenous
LCK	NM_005356.2	Endogenous
PRDM1	NM_001198.3	Endogenous
PPBP	NM_002704.2	Endogenous
PRKCD	NM_006254.3	Endogenous
PSMB10	NM_002801.2	Endogenous
PSMB5	NM_001130725.1	Endogenous
PSMB7	NM_002799.2	Endogenous
PSMB8	NM_004159.4	Endogenous
PSMB9	NM_002800.4	Endogenous
PSMC2	NM_002803.3	Endogenous
PSMD7	NM_002811.3	Endogenous
PTAFR	NM_000952.3	Endogenous
PTGER4	NM_000958.2	Endogenous
PTGS2	NM_000963.1	Endogenous
PTK2	NM_005607.3	Endogenous
PTPN2	NM_002828.2	Endogenous
PTPN22	NM_015967.4	Endogenous
PRF1	NM_005041.3	Endogenous
PTPRC_all	NM_080921.2	Endogenous
PUMA	NM_014417.2	Endogenous
PYCARD	NM_013258.3	Endogenous
RAF1	NM_002880.2	Endogenous
RAG1	NM_000448.2	Endogenous
RAG2	NM_000536.3	Endogenous
RARRES3	NM_004585.3	Endogenous
RELA	NM_021975.2	Endogenous
RELB	NM_006509.2	Endogenous
RORC	NM_001001523.1	Endogenous
RUNX1	NM_001754.4	Endogenous
S100A8	NM_002964.3	Endogenous
S100A9	NM_002965.2	Endogenous
S1PR1	NM_001400.3	Endogenous
SELE	NM_000450.2	Endogenous
SELL	NR_029467.1	Endogenous
SELPLG	NM_003006.3	Endogenous
SERPING1	NM_000062.2	Endogenous

SH2D1A	NM_001114937.2	Endogenous
SIGIRR	NM_021805.2	Endogenous
SKI	NM_003036.2	Endogenous
SLAMF1	NM_003037.2	Endogenous
SLAMF6	NM_001184714.1	Endogenous
SLAMF7	NM_021181.3	Endogenous
SLC2A1	NM_006516.2	Endogenous
SMAD3	NM_005902.3	Endogenous
SMAD5	NM_005903.5	Endogenous
SOCS1	NM_003745.1	Endogenous
SOCS3	NM_003955.3	Endogenous
SPP1	NM_000582.2	Endogenous
SRC	NM_005417.3	Endogenous
STAT1	NM_007315.2	Endogenous
STAT2	NM_005419.2	Endogenous
STAT3	NM_139276.2	Endogenous
STAT4	NM_003151.2	Endogenous
PTPN6	NM_002831.5	Endogenous
STAT5B	NM_012448.3	Endogenous
STAT6	NM_003153.3	Endogenous
STAT5A	NM_003152.2	Endogenous
TAGAP	NM_054114.3	Endogenous
TAL1	NM_003189.2	Endogenous
TAP1	NM_000593.5	Endogenous
TAP2	NM_000544.3	Endogenous
TAPBP	NM_003190.4	Endogenous
TBK1	NM_013254.2	Endogenous
TBX21	NM_013351.1	Endogenous
TCF4	NM_003199.1	Endogenous
TCF7	NM_003202.2	Endogenous
TFRC	NM_003234.1	Endogenous
TGFB1	NM_000660.3	Endogenous
TGFBI	NM_000358.2	Endogenous
TGFBR1	NM_004612.2	Endogenous
TGFBR2	NM_001024847.1	Endogenous
THY1	NM_006288.2	Endogenous
TICAM1	NM_014261.1	Endogenous
TIGIT	NM_173799.2	Endogenous
TIRAP	NM_148910.2	Endogenous
TLR1	NM_003263.3	Endogenous
TLR2	NM_003264.3	Endogenous
TLR3	NM_003265.2	Endogenous

TLR4	NM_138554.2	Endogenous
TLR5	NM_003268.3	Endogenous
TLR7	NM_016562.3	Endogenous
TLR8	NM_016610.2	Endogenous
TLR9	NM_017442.2	Endogenous
TMEM173	NM_198282.1	Endogenous
TNF	NM_000594.2	Endogenous
TNFAIP3	NM_006290.2	Endogenous
TNFAIP6	NM_007115.2	Endogenous
SYK	NM_003177.3	Endogenous
TNFRSF11A	NM_003839.2	Endogenous
TNFRSF13B	NM_012452.2	Endogenous
TNFRSF13C	NM_052945.3	Endogenous
TNFRSF14	NM_003820.2	Endogenous
TNFRSF17	NM_001192.2	Endogenous
TNFRSF1B	NM_001066.2	Endogenous
TNFRSF4	NM_003327.2	Endogenous
TNFRSF8	NM_152942.2	Endogenous
TNFRSF9	NM_001561.4	Endogenous
TNFSF10	NM_003810.2	Endogenous
TNFSF11	NM_003701.2	Endogenous
TNFSF12	NM_003809.2	Endogenous
TNFSF13B	NM_006573.4	Endogenous
TNFSF15	NM_001204344.1	Endogenous
TNFSF4	NM_003326.2	Endogenous
TNFSF8	NM_001244.3	Endogenous
TOLLIP	NM_019009.2	Endogenous
TP53	NM_000546.2	Endogenous
TRAF1	NM_005658.3	Endogenous
TRAF2	NM_021138.3	Endogenous
TRAF3	NM_145725.1	Endogenous
TRAF4	NM_004295.2	Endogenous
TRAF5	NM_004619.3	Endogenous
TRAF6	NM_145803.1	Endogenous
TYK2	NM_003331.3	Endogenous
UBE2L3	NM_198157.1	Endogenous
VCAM1	NM_001078.3	Endogenous
VTN	NM_000638.3	Endogenous
XBP1	NM_005080.2	Endogenous
XCL1	NM_002995.1	Endogenous
XCR1	NM_005283.2	Endogenous
ZAP70	NM_001079.3	Endogenous

ZBTB16	NM_006006.4	Endogenous
ZEB1	NM_001128128.1	Endogenous
sCTLA4	NM_001037631.1	Endogenous
ABCF1	NM_001090.2	Housekeeping
ALAS1	NM_000688.4	Housekeeping
EEF1G	NM_001404.4	Housekeeping
G6PD	NM_000402.2	Housekeeping
GAPDH	NM_002046.3	Housekeeping
GUSB	NM_000181.1	Housekeeping
HPRT1	NM_000194.1	Housekeeping
OAZ1	NM_004152.2	Housekeeping
POLR1B	NM_019014.3	Housekeeping
POLR2A	NM_000937.2	Housekeeping
PPIA	NM_021130.2	Housekeeping
RPL19	NM_000981.3	Housekeeping
SDHA	NM_004168.1	Housekeeping
TBP	NM_001172085.1	Housekeeping
TUBB	NM_178014.2	Housekeeping

Supplemental Table 4. Nanostring output

Probe Name	Accession #	II.2	HD average	StDev of HDs	II.2 vs. HD	DE Call of: II.2 vs. HD
ABCB1	NM_000927.3	8555,46	1218,05	110,53	7,02	Yes
ADA	NM_000022.2	899,96	1480,1	312,87	-1,64	Yes
AHR	NM_001621.3	5466,55	845,04	105,37	6,47	Yes
ARG2	NM_001172.3	571,03	379,45	34,72	1,5	Yes
ARHGDIB	NM_001175.4	24636,19	20015,79	1160,07	1,23	Yes
ATG12	NM_004707.2	738	200,84	25,63	3,67	Yes
ATG16L1	NM_198890.2	3783,5	1626,22	34,46	2,33	Yes
ATG5	NM_004849.2	3217,48	1291,41	120,05	2,49	Yes
ATG7	NM_001136031.2	48,42	406,14	51,24	-8,39	Yes
ATR	NM_001184.2	7742,33	1698,78	208,68	4,56	Yes
B2M	NM_004048.2	277605,06	123339,75	19412,68	2,25	Yes
B3GAT1	NM_018644.3	10,02	39,15	8,7	-3,91	Yes
BAD	NM_004322.3	190,34	95,72	37,45	1,99	Yes
BATF3	NM_018664.2	552,67	83,35	31,32	6,63	Yes
BAX	NM_138761.3	4650,07	2607,39	197,16	1,78	Yes
BCAP31	NM_005745.7	799,78	2757,2	117,98	-3,45	Yes
BCL10	NM_003921.2	1113,68	1601,62	309,83	-1,44	Yes
BCL2L11	NM_138621.4	455,82	148,21	32,26	3,08	Yes
BCL3	NM_005178.2	1541,12	187,17	55,23	8,23	Yes
BCL6	NM_001706.2	9946,31	2080,92	459,99	4,78	Yes
BID	NM_001196.2	4144,16	540,87	63,55	7,66	Yes
BLNK	NM_013314.2	140,25	287,83	163,56	-2,05	Yes
BRCA1	NM_007305.2	86,82	166,97	39,59	-1,92	Yes
BST2	NM_004335.2	46,75	149,49	25,81	-3,2	Yes
BTK	NM_000061.1	297,2	529,93	148,6	-1,78	Yes
BTLA	NM_181780.2	55,1	158,25	119,15	-2,87	Yes
C14orf166	NM_016039.2	3118,97	2453,15	263,3	1,27	Yes
C1QB	NM_000491.3	365,66	61,85	4,5	5,91	Yes
C1QBP	NM_001212.3	606,1	1600,2	253,84	-2,64	Yes
C6	NM_000065.2	701,27	25,17	7,21	27,86	Yes
CAMP	NM_004345.3	115,21	31,75	6,17	3,63	Yes
CASP1	NM_001223.3	724,64	1362,5	44,2	-1,88	Yes
CASP10	NM_032977.3	253,79	27,04	2,17	9,39	Yes
CASP2	NM_032982.2	4948,94	1913,04	89,23	2,59	Yes
CASP3	NM_032991.2	2593,02	553,42	137,7	4,69	Yes
CCBP2	NM_001296.3	106,86	34,21	8,04	3,12	Yes
CCL11	NM_002986.2	985,11	60,09	11,91	16,39	Yes
CCL15	NM_032965.3	722,97	35,28	8,27	20,49	Yes
CCL16	NM_004590.2	200,36	47,11	19,75	4,25	Yes
CCL18	NM_002988.2	768,05	52,89	8,42	14,52	Yes

CCL2	NM_002982.3	327,26	19,02	6,07	17,21	Yes
CCL20	NM_004591.1	116,88	19,97	6,18	5,85	Yes
CCL22	NM_002990.3	120,22	19,44	5,9	6,18	Yes
CCL3	NM_002983.2	1564,49	83,07	10,21	18,83	Yes
CCL4	NM_002984.2	11671,09	6839,19	1655,94	1,71	Yes
CCL5	NM_002985.2	75055,66	36522,25	5045,88	2,06	Yes
CCND3	NM_001760.2	1066,93	3877,95	665,52	-3,63	Yes
CCR1	NM_001295.2	437,46	277,4	97,29	1,58	Yes
CCR5	NM_000579.1	212,05	51,57	12,92	4,11	Yes
CCR6	NM_031409.2	1123,7	173,16	198,88	6,49	Yes
CCR8	NM_005201.2	85,15	33,17	10,1	2,57	Yes
CCRL1	NM_016557.2	631,14	110,92	28,99	5,69	Yes
CCRL2	NM_003965.4	200,36	58,37	14,09	3,43	Yes
CD14	NM_000591.2	178,66	269,6	200,82	-1,51	Yes
CD160	NM_007053.2	1938,5	2528,95	840,05	-1,3	Yes
CD163	NM_004244.4	125,23	45,36	15,2	2,76	Yes
CD164	NM_006016.4	7613,76	9902,05	272,36	-1,3	Yes
CD19	NM_001770.4	664,53	269,75	329,14	2,46	Yes
CD2	NM_001767.3	133,57	216,01	63,44	-1,62	Yes
CD209	NM_021155.2	355,64	22,38	8	15,89	Yes
CD22	NM_001771.2	337,28	591,12	1124,25	-1,75	Yes
CD24	NM_013230.2	150,27	41,59	18,91	3,61	Yes
CD244	NM_016382.2	661,2	2603,4	216,88	-3,94	Yes
CD247	NM_198053.1	29312,98	15434,43	987,69	1,9	Yes
CD27	NM_001242.4	166,97	581,12	509,92	-3,48	Yes
CD274	NM_014143.3	217,06	87,61	12,05	2,48	Yes
CD276	NM_001024736.1	198,69	48,24	15,01	4,12	Yes
CD28	NM_001243078.1	253,79	70,56	16,88	3,6	Yes
CD3EAP	NM_012099.1	108,53	32,25	6,7	3,37	Yes
CD40LG	NM_000074.2	1624,6	81,41	33,15	19,96	Yes
CD44	NM_001001392.1	8707,4	4970,16	110,45	1,75	Yes
CD45R0	NM_080921.3	240,43	414,63	110,45	-1,72	Yes
CD45RA	NM_002838.4	38340,96	15950,24	634,85	2,4	Yes
CD45RB	ENST00000367367.1	1003,48	4488,91	248,87	-4,47	Yes
CD46	NM_172350.1	1312,37	2707,23	286,72	-2,06	Yes
CD48	NM_001778.2	2816,76	7929,51	749,43	-2,82	Yes
CD53	NM_001040033.1	4379,58	12349,7	472,55	-2,82	Yes
CD58	NM_001779.2	111,87	50,78	8,66	2,2	Yes
CD59	NM_000611.4	1437,6	1037,95	363,46	1,39	Yes
CD6	NM_006725.3	2429,39	947,62	385,48	2,56	Yes
CD79A	NM_001783.3	255,46	1213,95	1694,01	-4,75	Yes
CD79B	NM_021602.2	432,45	186,31	68,59	2,32	Yes

CD82	NM_002231.3	128,57	78,17	25,12	1,64	Yes
CD83	NM_004233.3	7216,38	837,76	1146,59	8,61	Yes
CD86	NM_175862.3	1574,51	309,77	100,84	5,08	Yes
CD8A	NM_001768.5	751,36	1494,87	708,05	-1,99	Yes
CD8B	NM_004931.3	75,14	137,49	152,58	-1,83	Yes
CD96	NM_005816.4	1818,29	593,52	43,36	3,06	Yes
CD97	NM_078481.2	297,2	69,21	1,07	4,29	Yes
CD99	NM_002414.3	1075,28	2166,81	170,87	-2,02	Yes
CDKN1A	NM_000389.2	1868,38	380,3	69,43	4,91	Yes
CEBPB	NM_005194.2	297,2	741,18	38,58	-2,49	Yes
CFB	NM_001710.5	53,43	18,6	1,9	2,87	Yes
CFH	NM_001014975.2	166,97	68,89	51,38	2,42	Yes
CFI	NM_000204.3	457,49	32,88	3,07	13,92	Yes
CFP	NM_002621.2	95,17	175,37	45,96	-1,84	Yes
CHUK	NM_001278.3	2382,64	655,79	41,22	3,63	Yes
CIITA	NM_000246.3	529,29	183,09	404,73	2,89	Yes
CISH	NM_145071.2	202,03	84,67	17,58	2,39	Yes
CLEC7A	NM_197954.2	824,82	72,91	11,98	11,31	Yes
CMKLR1	NM_004072.1	183,67	2094,89	262,73	-11,41	Yes
CR1	NM_000651.4	240,43	722,91	384,3	-3,01	Yes
CR2	NM_001006658.1	133,57	261,3	369,26	-1,96	Yes
CRADD	NM_003805.3	1319,05	135,49	8,5	9,74	Yes
CSF1	NM_000757.4	335,61	133,9	37,62	2,51	Yes
CSF1R	NM_005211.2	145,26	385,37	96,39	-2,65	Yes
CSF2	NM_000758.2	136,91	22,36	2,62	6,12	Yes
CSF2RB	NM_000395.2	706,28	411,7	216,27	1,72	Yes
CSF3R	NM_156038.2	36,73	285,55	187,55	-7,77	Yes
CTSC	NM_001814.4	2382,64	5332,91	262,64	-2,24	Yes
CTSG	NM_001911.2	60,11	24,74	7,36	2,43	Yes
CTSS	NM_004079.3	7371,66	10200,31	900,96	-1,38	Yes
CX3CR1	NM_001337.3	267,15	3207,28	171,75	-12,01	Yes
CXCL1	NM_001511.1	308,89	39,72	23,61	7,78	Yes
CXCL10	NM_001565.1	106,86	18,52	4,92	5,77	Yes
CXCL2	NM_002089.3	682,9	47,87	33,1	14,27	Yes
CXCL9	NM_002416.1	113,54	20,82	3,89	5,45	Yes
CXCR1	NM_000634.2	492,56	121,59	17,43	4,05	Yes
CXCR2	NM_001557.2	245,44	829,37	227,02	-3,38	Yes
CXCR3	NM_001504.1	1958,54	215,79	50,56	9,08	Yes
CXCR4	NM_003467.2	13556,17	9897,47	1475,73	1,37	Yes
CXCR6	NM_006564.1	392,38	147,36	37,45	2,66	Yes
CYBB	NM_000397.3	727,98	2676,86	707,11	-3,68	Yes
Cyclin E	NM_001238.1	312,23	166,13	42,8	1,88	Yes

DEFB1	NM_005218.3	155,28	53,5	8,45	2,9	Yes
DEFB103A	NM_001081551.2	310,56	30,98	10,45	10,03	Yes
DUSP4	NM_057158.2	2279,12	72,13	37,73	31,6	Yes
E2F1	NM_005225.1	325,59	161,14	50,36	2,02	Yes
EGR1	NM_001964.2	1120,36	757,06	41,06	1,48	Yes
EGR2	NM_000399.3	98,51	37,12	5,47	2,65	Yes
ENTPD1	NM_001098175.1	2441,08	493,38	165,14	4,95	Yes
ETS1	NM_005238.3	7354,96	18704,29	1496,29	-2,54	Yes
FADD	NM_003824.2	175,32	114,31	9,1	1,53	Yes
FAS	NM_000043.3	73,47	18,71	6,36	3,93	Yes
FCAR	NM_133280.1	48,42	17,89	1,85	2,71	Yes
FCER1A	NM_002001.2	28,38	1095,06	526,42	-38,58	Yes
FCER1G	NM_004106.1	10921,41	20397,66	1286,44	-1,87	Yes
FCGR1A/B	NM_000566.3	161,96	49,29	1,31	3,29	Yes
FCGR2A	NM_021642.3	81,81	199,21	30,53	-2,43	Yes
FCGR2A/C	NM_201563.4	425,77	1193,36	135,36	-2,8	Yes
FCGR2B	NM_001002273.1	43,41	100,73	58,29	-2,32	Yes
FCGR3A/B	NM_000570.4	5009,05	23174,2	1408,81	-4,63	Yes
FKBP5	NM_001145775.1	175,32	1201,21	121,29	-6,85	Yes
GADD45A	NM_001924.2	996,8	676,23	17,22	1,47	Yes
GATA3	NM_001002295.1	300,54	141,77	23,48	2,12	Yes
GBP1	NM_002053.1	1547,8	627,65	46,15	2,47	Yes
GBP5	NM_052942.3	130,24	339,61	181,53	-2,61	Yes
GFI1	NM_005263.2	7837,5	1222,5	290,13	6,41	Yes
GNLY	NM_006433.2	64132,58	49382,47	328,12	1,3	Yes
GPR183	NM_004951.3	13273,99	2748,15	746,36	4,83	Yes
GZMB	NM_004131.3	3942,12	7930,39	2250,83	-2,01	Yes
HAVCR2	NM_032782.3	1746,49	2237,97	548,74	-1,28	Yes
HFE	NM_139011.2	283,85	53,98	9,07	5,26	Yes
HLA-A	NM_002116.5	11545,87	9322,5	2131,77	1,24	Yes
HLA-B	NM_005514.6	61898,54	41438,44	3244,81	1,49	Yes
HLA-DMB	NM_002118.3	773,06	322,57	121,71	2,4	Yes
HLA-DOB	NM_002120.3	884,93	632,47	498,38	1,4	Yes
HLA-DPB1	NM_002121.4	7172,96	2077,55	4172,79	3,45	Yes
HLA-DQB1	NM_002123.3	30,05	80,17	487,05	-2,67	Yes
HLA-DRA	NM_019111.3	10737,74	14771,74	9660,82	-1,38	Yes
HLA-DRB1	NM_002124.2	485,88	226,15	672,7	2,15	Yes
HLA-DRB3	NM_022555.3	452,48	248,2	625,41	1,82	Yes
ICAM1	NM_000201.2	4294,43	598,74	88,74	7,17	Yes
ICAM2	NM_000873.3	666,2	3762,83	629,51	-5,65	Yes
ICAM3	NM_002162.3	1512,73	2437,89	164,82	-1,61	Yes
ICOSLG	NM_015259.4	983,44	109,49	104,22	8,98	Yes

IDO1	NM_002164.3	586,06	32,67	2,64	17,94	Yes
IFI16	NM_005531.1	639,49	1893,74	60,22	-2,96	Yes
IFIH1	NM_022168.2	606,1	249,83	45,58	2,43	Yes
IFIT2	NM_001547.4	2115,49	602,67	115,88	3,51	Yes
IFITM1	NM_003641.3	49417,65	65216,93	8267,61	-1,32	Yes
IFNA2	NM_000605.3	980,1	48,05	23,7	20,4	Yes
IFNAR2	NM_000874.3	1567,83	2747,68	257,2	-1,75	Yes
IFNB1	NM_002176.2	2731,6	24,05	7,02	113,58	Yes
IFNG	NM_000619.2	225,41	110,62	36,67	2,04	Yes
IGF2R	NM_000876.1	2541,26	3681,87	651,55	-1,45	Yes
IKBKAP	NM_003640.3	105,19	510,6	32,24	-4,85	Yes
IKBKB	NM_001556.1	382,36	608	45,92	-1,59	Yes
IKZF1	NM_006060.3	4027,28	2723,36	326,63	1,48	Yes
IKZF2	NM_016260.2	589,4	1158,84	151,46	-1,97	Yes
IKZF3	NM_183232.2	8320,04	2952,6	495,31	2,82	Yes
IL10RA	NM_001558.2	1644,64	4121,05	588,11	-2,51	Yes
IL12B	NM_002187.2	487,55	37,03	5,04	13,17	Yes
IL12RB1	NM_005535.1	338,95	1215,07	288,56	-3,58	Yes
IL13RA1	NM_001560.2	46,75	146,48	97,87	-3,13	Yes
IL15	NM_172174.1	2000,28	221,01	52,33	9,05	Yes
IL17F	NM_052872.3	121,89	27,61	6,15	4,41	Yes
IL18	NM_001562.2	981,77	149,71	26,5	6,56	Yes
IL18R1	NM_003855.2	894,95	1643,51	507,46	-1,84	Yes
IL18RAP	NM_003853.2	21418,71	6442,07	1912,43	3,32	Yes
IL1B	NM_000576.2	1036,87	131,02	79,35	7,91	Yes
IL1R2	NM_173343.1	263,81	127,06	26,32	2,08	Yes
IL1RN	NM_000577.3	387,37	67,57	26,57	5,73	Yes
IL20	NM_018724.3	165,3	19,74	2,76	8,38	Yes
IL21R	NM_021798.2	3227,5	842,46	202,78	3,83	Yes
IL23A	NM_016584.2	225,41	97,11	19	2,32	Yes
IL28A/B	NM_172139.2	115,21	35,61	0,85	3,24	Yes
IL2RB	NM_000878.2	62102,24	23537,61	4177,83	2,64	Yes
IL2RG	NM_000206.1	17394,77	24899,1	900,27	-1,43	Yes
IL3	NM_000588.3	88,49	21,37	0,51	4,14	Yes
IL32	NM_001012633.1	6545,16	283,46	83,95	23,09	Yes
IL4R	NM_000418.2	6874,09	2126,35	1488,14	3,23	Yes
IL6R	NM_000565.2	115,21	248,68	75,54	-2,16	Yes
IL6ST	NM_002184.2	1450,96	471,46	31,46	3,08	Yes
IL7	NM_000880.2	390,71	87,12	48,39	4,48	Yes
IL8	NM_000584.2	158,62	32,26	23,07	4,92	Yes
ILF3	NM_001137673.1	5312,94	1348,96	310,15	3,94	Yes
IRAK2	NM_001570.3	288,86	200,29	49,89	1,44	Yes

IRF1	NM_002198.1	2088,78	1302,57	176,58	1,6	Yes
IRF3	NM_001571.5	200,36	461,14	11,94	-2,3	Yes
IRF4	NM_002460.1	1006,82	718,29	291,48	1,4	Yes
IRF8	NM_002163.2	971,76	355,84	150,13	2,73	Yes
IRGM	NM_001145805.1	1158,76	100,09	26,57	11,58	Yes
ITGA2B	NM_000419.3	146,93	76,06	10,75	1,93	Yes
ITGA5	NM_002205.2	1587,87	977,26	173,42	1,62	Yes
ITGA6	NM_000210.1	297,2	2166,35	588,16	-7,29	Yes
ITGAE	NM_002208.4	6688,76	1369,45	173,83	4,88	Yes
ITGAL	NM_002209.2	13925,17	17715,55	713,06	-1,27	Yes
ITGAM	NM_000632.3	9465,44	5969,57	1306,25	1,59	Yes
ITGAX	NM_000887.3	1467,65	1927,02	265,06	-1,31	Yes
ITGB2	NM_000211.2	1091,97	3144,56	726,21	-2,88	Yes
ITLN1	NM_017625.2	166,97	35,75	10,4	4,67	Yes
JAK2	NM_004972.2	166,97	380,18	23,12	-2,28	Yes
JAK3	NM_000215.2	467,51	902,05	116,12	-1,93	Yes
KIR3DL1	NM_013289.2	647,84	1087,98	1281,92	-1,68	Yes
KIR3DL2	NM_006737.2	1442,61	2798,51	107,78	-1,94	Yes
KIR_Activating_Subgroup_1	NM_001083539.1	801,45	271,85	518,77	2,95	Yes
KIR_Activating_Subgroup_2	NM_014512.1	1225,55	534,64	626,48	2,29	Yes
KIR_Inhibiting_Subgroup_2	NM_014511.3	904,97	695,54	430,53	1,3	Yes
KIT	NM_000222.2	407,4	243,84	4,78	1,67	Yes
KLRB1	NM_002258.2	142729,64	29905,44	2653,36	4,77	Yes
KLRC1	NM_002259.3	1182,14	825,34	455,11	1,43	Yes
KLRC2	NM_002260.3	1377,49	690,81	523,3	1,99	Yes
KLRC3	NM_007333.2	1147,07	1475,83	724,25	-1,29	Yes
KLRC4	NM_013431.2	637,82	1169,87	214,05	-1,83	Yes
KLRD1	NM_002262.3	1983,59	15974,73	1512,1	-8,05	Yes
KLRG1	NM_005810.3	106,86	841,4	224,29	-7,87	Yes
KLRG2	NM_198508.2	243,77	38,31	2,4	6,36	Yes
KLRK1	NM_007360.1	158,62	59,66	14,87	2,66	Yes
LAIR1	NM_002287.3	1794,91	4774,28	499,27	-2,66	Yes
LAMP3	NM_014398.3	732,99	31,54	6,02	23,24	Yes
LCK	NM_005356.2	721,3	5074,53	468,75	-7,04	Yes
LCP2	NM_005565.3	2569,64	5045,35	141,85	-1,96	Yes
LILRA2	NM_006866.2	285,52	144,97	25,03	1,97	Yes
LILRA3	NM_006865.3	230,42	78,58	50,28	2,93	Yes
LILRA4	NM_012276.3	193,68	58,95	8,23	3,29	Yes
LILRA5	NM_181879.2	664,53	104,98	61,11	6,33	Yes
LILRA6	NM_024318.2	1604,57	96,17	22,47	16,69	Yes
LILRB1	NM_001081637.1	202,03	581,1	259,46	-2,88	Yes
LILRB2	NM_005874.1	160,29	321,86	77,25	-2,01	Yes

LILRB4	NM_001081438.1	509,25	128,82	134,6	3,95	Yes
LILRB5	NM_001081442.1	123,56	26,9	7,73	4,59	Yes
LTA	NM_000595.2	854,88	139,66	57,12	6,12	Yes
LTBR	NM_002342.1	225,41	40,9	2,11	5,51	Yes
MAF	NM_005360.4	173,65	97,28	5,82	1,78	Yes
MALT1	NM_006785.2	1165,44	1920,81	375,39	-1,65	Yes
MAP4K1	NM_007181.3	1577,85	2324,82	470,89	-1,47	Yes
MAP4K2	NM_004579.2	4534,86	1102,04	269,16	4,11	Yes
MAPK11	NM_002751.5	482,54	74,91	5,07	6,44	Yes
MAPK14	NM_001315.1	913,32	668	63,05	1,37	Yes
MAPKAPK2	NM_004759.3	2060,39	1133,8	127,22	1,82	Yes
MARCO	NM_006770.3	103,52	25,86	2,73	4	Yes
MASP1	NM_139125.3	405,73	54,37	4,8	7,46	Yes
MBL2	NM_000242.2	75,14	25,84	1,65	2,91	Yes
MBP	NM_002385.2	784,75	299,73	27,73	2,62	Yes
MDM2	NM_001145337.1	540,98	294,27	83,22	1,84	Yes
MME	NM_000902.2	285,52	70,76	27,2	4,04	Yes
MR1	NM_001531.2	646,17	389,73	72,34	1,66	Yes
MS4A1	NM_152866.2	362,32	2057,97	3583,28	-5,68	Yes
MYD88	NM_002468.3	11358,86	3700,08	177,69	3,07	Yes
NCAM1	NM_000615.5	1450,96	2956,94	492,11	-2,04	Yes
NCR1	NM_004829.5	2801,73	5576,72	1508,56	-1,99	Yes
NFATC2	NM_012340.3	9657,46	7695,73	547	1,25	Yes
NFATC3	NM_004555.2	5883,97	9586,43	1204,13	-1,63	Yes
NFIL3	NM_005384.2	941,7	1884,25	247,4	-2	Yes
NFKB1	NM_003998.2	16050,68	690,62	45,83	23,24	Yes
NFKB2	NM_002502.2	535,97	32,47	10,84	16,51	Yes
NFKBIA	NM_020529.1	851,54	112,96	15,91	7,54	Yes
NOD2	NM_022162.1	1257,27	232,18	32,38	5,41	Yes
NOTCH1	NM_017617.3	1028,53	765,93	224,14	1,34	Yes
PAX5	NM_016734.1	1105,33	736,42	1504,16	1,5	Yes
PDGFRB	NM_002609.3	215,39	502,08	130,96	-2,33	Yes
PECAM1	NM_000442.3	4079,04	3018,91	273,05	1,35	Yes
PML	NM_002675.3	514,26	934,45	338,69	-1,82	Yes
POU2F2	NM_002698.2	182	558,32	250,02	-3,07	Yes
PPARG	NM_015869.3	161,96	31,85	2,1	5,09	Yes
PPBP	NM_002704.2	2374,29	85,79	168,92	27,67	Yes
PRDM1	NM_001198.3	2325,87	4293,02	742,96	-1,85	Yes
PRF1	NM_005041.3	11948,26	54719,07	6921,39	-4,58	Yes
PSMB10	NM_002801.2	1349,1	2387,92	233,97	-1,77	Yes
PSMB7	NM_002799.2	5369,71	3480,02	399,31	1,54	Yes
PSMB9	NM_002800.4	2608,05	6259,75	1316,6	-2,4	Yes

PSMC2	NM_002803.3	5919,03	2135,85	133,47	2,77	Yes
PSMD7	NM_002811.3	9640,76	3537,01	257,89	2,73	Yes
PTAFR	NM_000952.3	520,94	101,93	63,07	5,11	Yes
PTGER4	NM_000958.2	235,43	144,57	17,41	1,63	Yes
PTGS2	NM_000963.1	190,34	126,61	187,7	1,5	Yes
PTK2	NM_005607.3	956,73	259,95	89,83	3,68	Yes
PTPN2	NM_002828.2	235,43	137,65	11,37	1,71	Yes
PTPN22	NM_015967.4	1374,15	2199,84	524,04	-1,6	Yes
PTPN6	NM_002831.5	941,7	2930,15	771,92	-3,11	Yes
PTPRC_all	NM_080921.2	2327,54	1270,29	64,19	1,83	Yes
PUMA	NM_014417.2	101,85	48,68	4,28	2,09	Yes
PYCARD	NM_013258.3	71,8	305,97	39,88	-4,26	Yes
RAF1	NM_002880.2	2943,65	1540,12	6,78	1,91	Yes
RAG1	NM_000448.2	110,2	60,8	8,11	1,81	Yes
RARRES3	NM_004585.3	5733,7	8948,66	1381,71	-1,56	Yes
RELA	NM_021975.2	1369,14	698,86	120,63	1,96	Yes
RELB	NM_006509.2	303,88	105,01	12,02	2,89	Yes
RORC	NM_001001523.1	120,22	70,17	7,82	1,71	Yes
RUNX1	NM_001754.4	277,17	740,49	121,91	-2,67	Yes
S100A8	NM_002964.3	367,33	1281,54	1010,36	-3,49	Yes
SELL	NR_029467.1	4406,3	10571,6	709,35	-2,4	Yes
SELPLG	NM_003006.3	150,27	451,13	30,03	-3	Yes
SERPING1	NM_000062.2	784,75	147,28	9,24	5,33	Yes
SH2D1A	NM_001114937.2	2856,83	1608,03	460,41	1,78	Yes
SIGIRR	NM_021805.2	936,69	2192,36	679,78	-2,34	Yes
SKI	NM_003036.2	17047,48	4538,79	706,25	3,76	Yes
SLAMF6	NM_001184714.1	125,23	1094,13	159,2	-8,74	Yes
SLAMF7	NM_021181.3	3654,94	5060,82	635,64	-1,38	Yes
SMAD5	NM_005903.5	2249,06	1052,28	89,94	2,14	Yes
SOCS3	NM_003955.3	136,91	63,88	8,42	2,14	Yes
SPP1	NM_000582.2	230,42	37,07	8,78	6,22	Yes
SRC	NM_005417.3	90,16	36,73	5,87	2,45	Yes
STAT2	NM_005419.2	1300,68	2350,05	150,98	-1,81	Yes
STAT3	NM_139276.2	1763,19	2639,15	80,01	-1,5	Yes
STAT4	NM_003151.2	1315,71	1978,04	455,67	-1,5	Yes
STAT5A	NM_003152.2	2616,4	806,45	46,93	3,24	Yes
STAT5B	NM_012448.3	242,1	527,28	116,37	-2,18	Yes
STAT6	NM_003153.3	514,26	4977,17	723,25	-9,68	Yes
SYK	NM_003177.3	753,03	2461,64	867,37	-3,27	Yes
TAGAP	NM_054114.3	7855,87	4085,88	1220,67	1,92	Yes
TAL1	NM_003189.2	374,01	99,44	38,81	3,76	Yes
TAP1	NM_000593.5	1268,96	905,09	173,57	1,4	Yes

TAPBP	NM_003190.4	1345,77	2055,44	182,74	-1,53	Yes
TBK1	NM_013254.2	1586,2	609,65	26,81	2,6	Yes
TBX21	NM_013351.1	6812,31	10490,53	2202,93	-1,54	Yes
TCF4	NM_003199.1	1210,52	874,17	332,78	1,38	Yes
TCF7	NM_003202.2	5560,05	2932,14	193,28	1,9	Yes
TFRC	NM_003234.1	2385,98	605,69	174,9	3,94	Yes
TGFB1	NM_000660.3	13891,77	6230,58	1586,88	2,23	Yes
TGFBI	NM_000358.2	222,07	688,63	359,53	-3,1	Yes
TGFBR1	NM_004612.2	2062,06	3371,22	508,81	-1,63	Yes
TGFBR2	NM_001024847.1	1031,86	683,42	145,34	1,51	Yes
TIRAP	NM_148910.2	298,87	134	25,5	2,23	Yes
TLR3	NM_003265.2	445,81	311,51	9,27	1,43	Yes
TLR4	NM_138554.2	178,66	111,56	17,09	1,6	Yes
TLR7	NM_016562.3	866,57	88,11	29,53	9,84	Yes
TLR8	NM_016610.2	123,56	36,71	10,14	3,37	Yes
TLR9	NM_017442.2	581,05	192,31	54,05	3,02	Yes
TMEM173	NM_198282.1	141,92	726,56	156,05	-5,12	Yes
TNF	NM_000594.2	268,82	124,88	34,55	2,15	Yes
TNFAIP3	NM_006290.2	42383,27	9845,48	418,06	4,3	Yes
TNFRSF10C	NM_003841.3	36,73	200,6	158,18	-5,46	Yes
TNFRSF13B	NM_012452.2	106,86	218,41	119,74	-2,04	Yes
TNFRSF14	NM_003820.2	267,15	503,09	219,55	-1,88	Yes
TNFRSF17	NM_001192.2	472,52	1410	2683,39	-2,98	Yes
TNFRSF1B	NM_001066.2	2992,07	1243,34	291,12	2,41	Yes
TNFRSF8	NM_152942.2	519,27	102,02	17,83	5,09	Yes
TNFSF10	NM_003810.2	1796,58	774,31	140,91	2,32	Yes
TNFSF12	NM_003809.2	76,81	366,87	126,97	-4,78	Yes
TNFSF13B	NM_006573.4	714,62	431,9	63,86	1,65	Yes
TNFSF15	NM_001204344.1	716,29	52,37	6,49	13,68	Yes
TNFSF4	NM_003326.2	1190,48	144,31	25,33	8,25	Yes
TOLLIP	NM_019009.2	557,67	328,22	78,21	1,7	Yes
TP53	NM_000546.2	549,33	1390,08	171	-2,53	Yes
TRAF1	NM_005658.3	689,58	358,18	79,96	1,93	Yes
TRAF2	NM_021138.3	358,98	156,52	14,91	2,29	Yes
TRAF3	NM_145725.1	2487,83	428,72	161,99	5,8	Yes
TRAF4	NM_004295.2	3958,82	625,44	181,86	6,33	Yes
TRAF6	NM_145803.1	2499,52	748,33	81,06	3,34	Yes
TYK2	NM_003331.3	724,64	1179,39	402,82	-1,63	Yes
UBE2L3	NM_198157.1	768,05	2311,61	283,19	-3,01	Yes
VCAM1	NM_001078.3	1225,55	24,51	8,33	50	Yes
VTN	NM_000638.3	83,48	22,57	3,44	3,7	Yes
XBP1	NM_005080.2	2033,68	3156,85	1006,48	-1,55	Yes

XCL1	NM_002995.1	5499,94	144,29	13,69	38,12	Yes
XCR1	NM_005283.2	392,38	138,18	71,81	2,84	Yes
ZAP70	NM_001079.3	2357,59	2968,22	867,17	-1,26	Yes
ZBTB16	NM_006006.4	5301,25	2460,07	213,42	2,15	Yes
ZEB1	NM_001128128.1	1245,58	638,61	51,35	1,95	Yes

Supplemental Table 5. Full list of pathways

GO.ID	Description	p.Val	Genes
GO:0070098	chemokine-mediated signaling pathway	1.88E-03	CXCL2,CCL2,XCL1,PPBP,CCL11,CCL18, CCL15,CCL3
GO:1990868	response to chemokine	1.88E-03	CXCL2,CCL2,XCL1,PPBP,CCL11,CCL18, CCL15,CCL3
GO:1990869	cellular response to chemokine	1.88E-03	CXCL2,CCL2,XCL1,PPBP,CCL11,CCL18,CCL15,CCL3
REAC:R-HSA-6783783	Interleukin-10 signaling	4.98E-03	CXCL2,ICAM1,CCL22,CCL2,IL12B,CD86,CCL20,IL1B,IL1RN, IL18,CXCL1,CSF2,CXCL10,PTAFR,IL8,CCL3
GO:0030593	neutrophil chemotaxis	6.76E-03	CXCL2,CCL2,XCL1,PPBP,CCL11,CCL18,CCL15,CCL3
GO:0071621	granulocyte chemotaxis	1.03E-02	CXCL2,CCL2,XCL1,PPBP,CCL11,CMKLR1,CCL18,CCL15, CCL3
GO:1990266	neutrophil migration	1.06E-02	CXCL2,CCL2,XCL1,PPBP,CCL11,CCL18,CCL15,CCL3
GO:0097530	granulocyte migration	1.98E-02	CXCL2,CCL2,XCL1,PPBP,CCL11,CCL18,CCL15,CCL3
GO:0097529	myeloid leukocyte	4.19E-02	CXCL2,CCL2,XCL1,PPBP,CX3CR1,CCL11,CMKLR1,CCL18, CCL15,CCL3
REAC:R-HSA-500792	GPCR ligand binding	4.40E-02	CXCL2,CCR6,CCL20,CD97,CXCL9,XCL1,CCR5,CXCR1, PPBP,CXCL1,CX3CR1,CXCL10,PTAFR,IL8,GPR183,CMKLR 1, CXCR3,CCL16
REAC:R-HSA-380108	Chemokine receptors bind chemokines	4.61E-02	CXCL2,CCR6,CCL20,CXCL9,XCL1,CCR5,CXCR1,PPBP, CXCL1,CX3CR1,CXCL10,IL8,CXCR3,CCL16

Supplemental Table 6. List of antibodies

Experiment	Marker	Fluorophore	Clone	Source
NK differentiation panel	CD34	PE	561	Biolegend
	CD56	BV421	HCD56	Biolegend
	CD16	PE-TEXAS RED	3G8	Invitrogen
	CD45	BUV395	H130	BD Biosciences
	CD117	PE-CY7	A3C6E2	Biolegend
	CD94	APC	DX22	Biolegend
	CD3	BV711	SK7	Biolegend
NK panel	CD56	QD605	HCD56	Biolegend
	CD16	PE-TEXAS RED	3G8	Biolegend
	CD3	BV711	SK7	Biolegend
NK activation panel	CD107a	APC	H4A3	Biolegend
	CD16	PE-CF594	3G8	BD Biosciences
	CD56	PeCy7	NCAM16.2	BD Biosciences
	CD3	V500	UCHT1	BD Biosciences
	IFN-γ	AF700	B27	Biolegend
	Perforin	BV421	dG9	Biolegend
NK maturation panel	CD14	AF700	63D3	Biolegend
	CD16	PE-CF594	3G8	BD Biosciences
	CD56	PE-Cy7	NCAM16.2	BD Biosciences
	CD3	V500	UCHT1	BD Biosciences
	CXCR1	PerCP/Cy5.5	8F1/CXCR1	Biolegend
	CXCR3	BV650	G025H7	Biolegend
	CX3CR1	APC	2A9-1	Biolegend
	CD94	FITC	DX22	Biolegend
	CD57	PB	HNK-1	Biolegend
	CMKLR1	PE	84939	R&D Systems
Memory T cells panel	CD4	PE/Cy5	RPA-T4	Biolegend
	CD3	PE	BW264/56	Miltenyi Biotec
	CD8	V450	RPA-T8	BD Biosciences
	CD27	APC/Cy7	O323	Biolegend
	CD45RA	FITC	HI100	BD Biosciences
T cells subsets panel	CD3	V500	UCHT1	BD Biosciences
	CD19	APCH7	SJ25C1	BD Biosciences
	CD8a	BV785	RPA-T8	Biolegend
	TCR Vα7.2	PE	3C10	Biolegend
	CD161	APC	HP-3G10	Invitrogen
	TCRγδ	FITC	B1	BD Biosciences

Western blot	GINS4		Rabbit polyclonal	Abcam ab101346
	GINS1		EPR13359	Abcam ab181112
	GINS2		Rabbit polyclonal	proteintech 16247-1ap
	GINS3		Rabbit polyclonal	proteintech 15651-1-ap
	ACTIN		Rabbit polyclonal	Sigma A5060
	phospho-CHK1		d12h3	cell signaling 12302s
	CHK1		2G1D5	cell signaling 2360s
	ubPCNA		D5C7P	cell signaling 13439s
	PCNA		Rabbit polyclonal	abcam 18197
	Goat anti-mouse	IRDye 680RD		Licor 926-68070
	Goat anti-rabbit	IRDye 800CW		Licor 926-32211

Supplemental Table 7. List of taqman assays

Taqman assay	Assay cod	Source
GINS4	HS01077879_m1	Thermofisher
GINS1	Hs01040834_m1	Thermofisher
GINS2	Hs00211479_m1	Thermofisher
GINS3	Hs01090589_m1	Thermofisher
CX3CR1	Hs01598433_m1	Thermofisher
CMKLR1	HS01386064_m1	Thermofisher
CXCR1	Hs01921207_s1	Thermofisher
CXCR3	Hs00171041_m1	Thermofisher
HPRT1	HS02800695_m1	Thermofisher

Full unedited gels

Conte et al.

Full unedited gel for Figure 3A

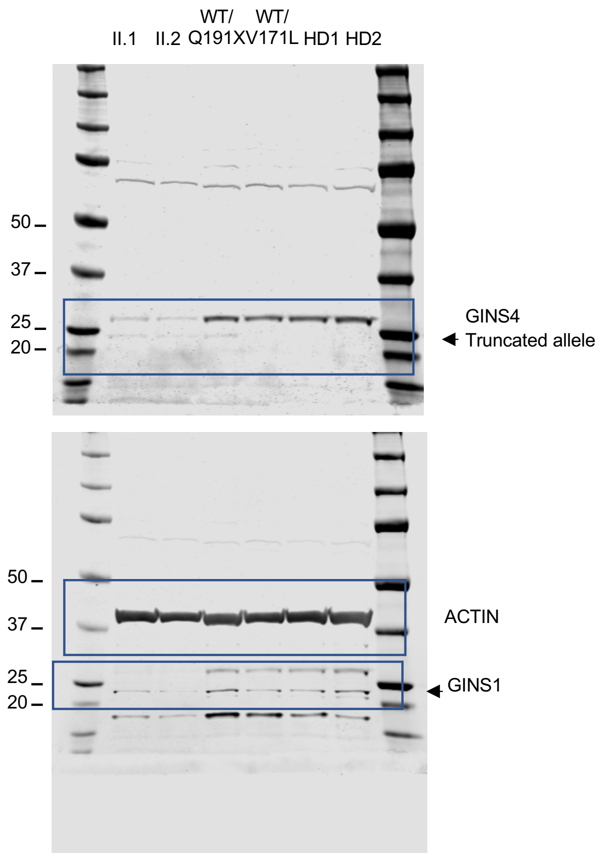

Full unedited gel for Figure 3C

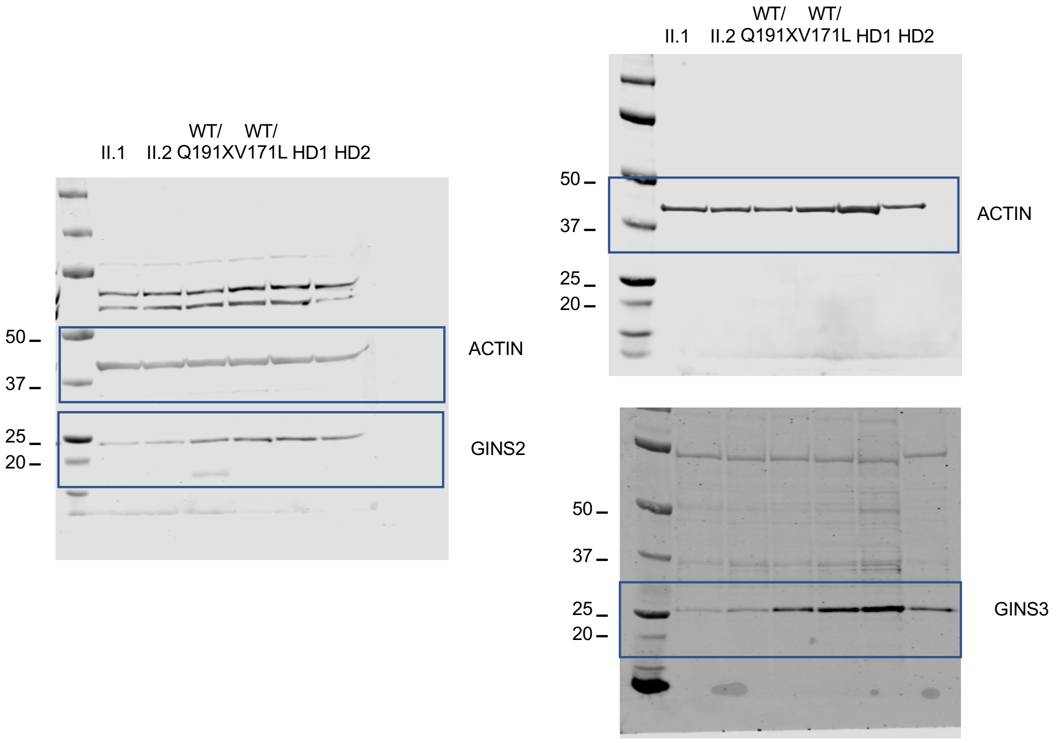

Full unedited gel for Figure 3D

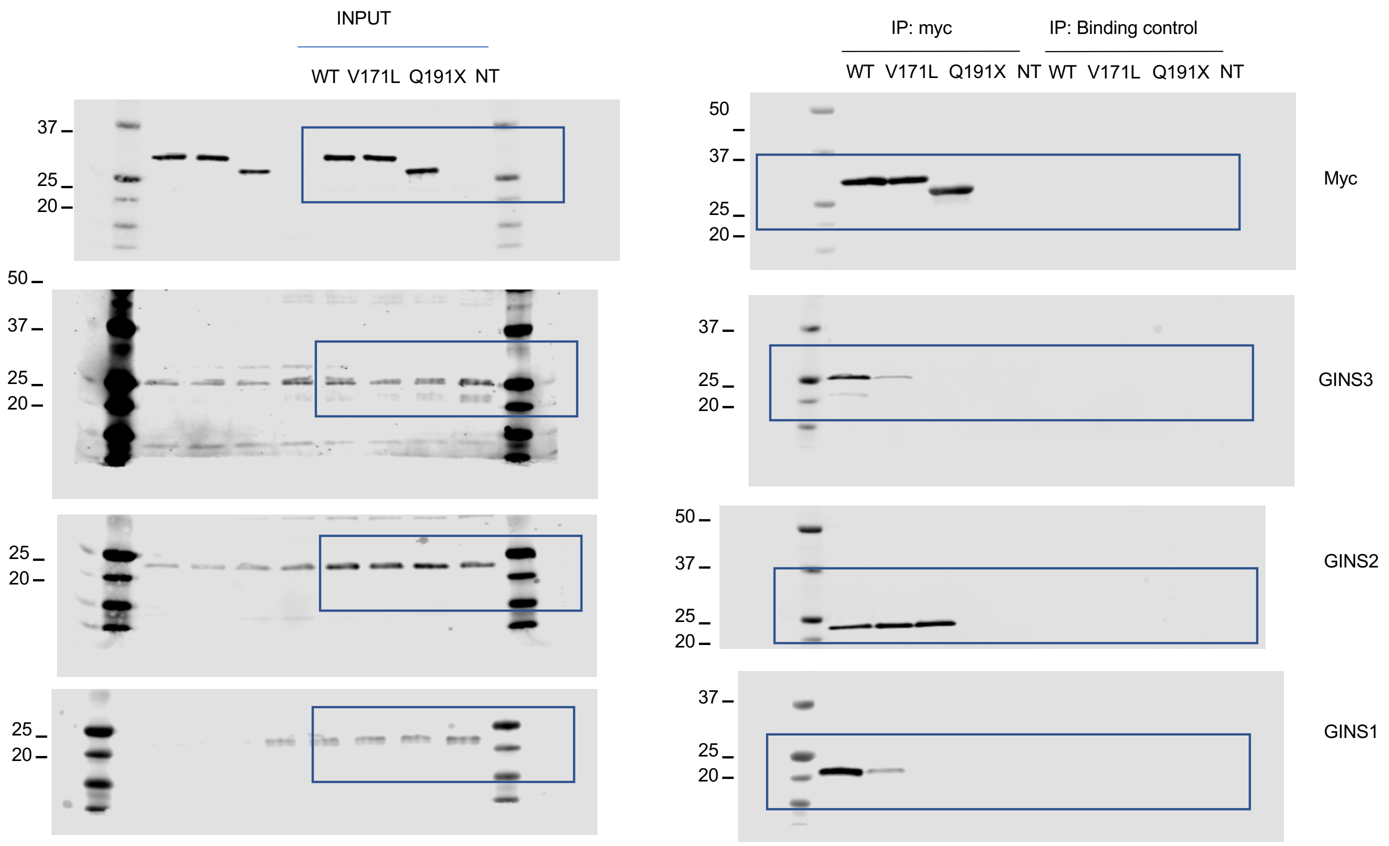

Full unedited gel for Figure 4E

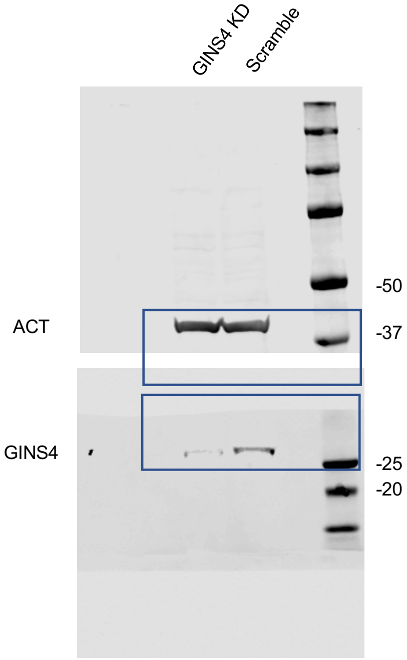

Full unedited gel for Supplemental figure 6

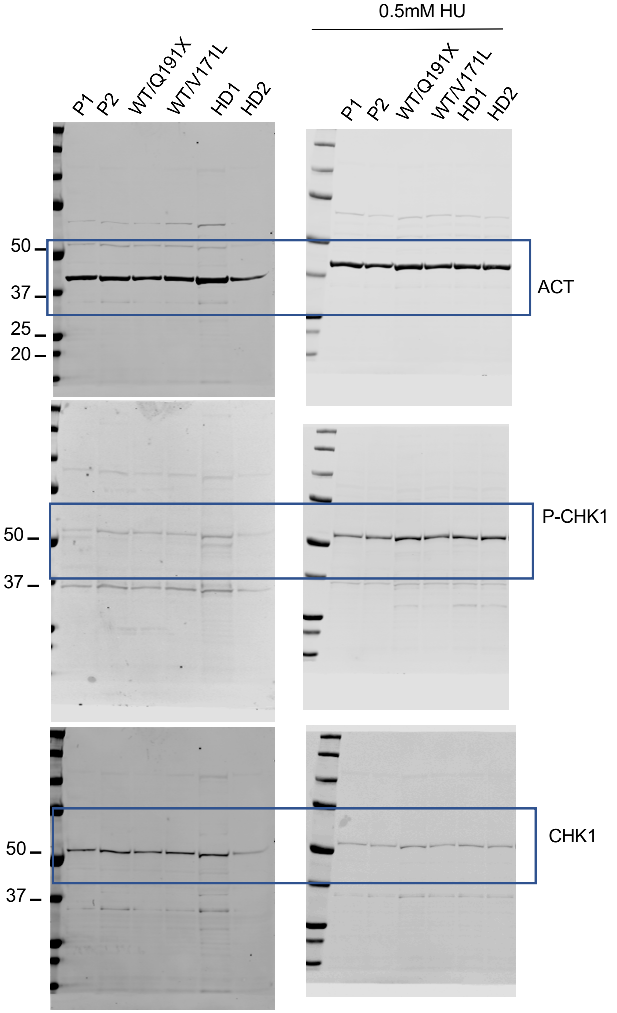

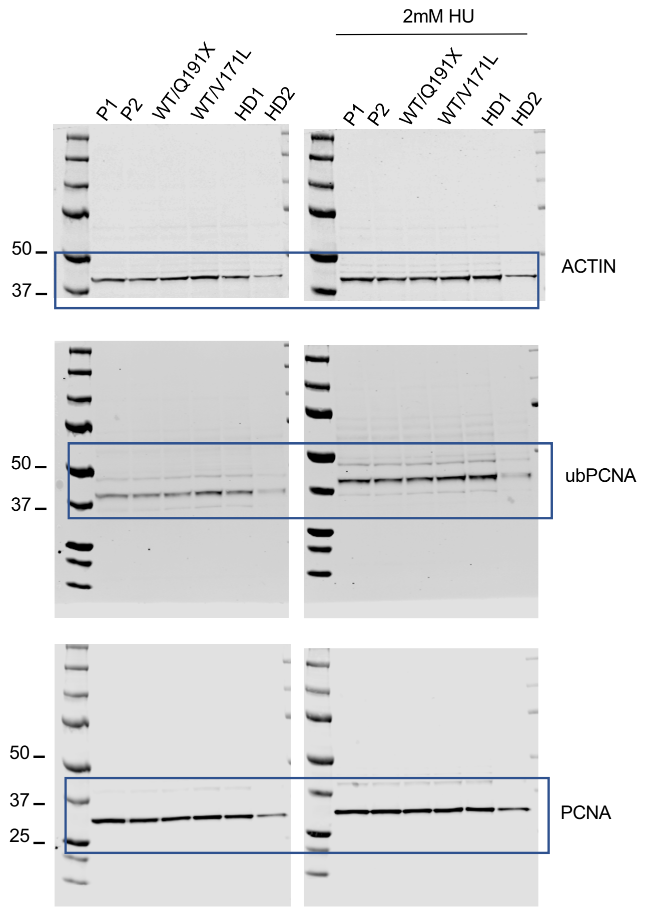

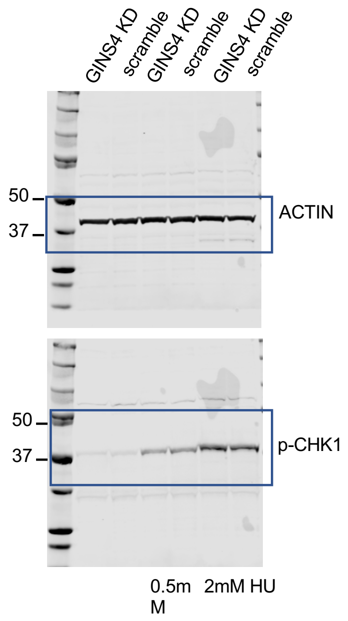

Supplement: Supplemental data [file jciinsight-7-154948-s241.pdf]
